# Releasing chemical energy in spatially programmed ferroelectrics

**DOI:** 10.1038/s41467-022-34819-z

**Published:** 2022-11-15

**Authors:** Yong Hu, Jennifer L. Gottfried, Rose Pesce-Rodriguez, Chi-Chin Wu, Scott D. Walck, Zhiyu Liu, Sangeeth Balakrishnan, Scott Broderick, Zipeng Guo, Qiang Zhang, Lu An, Revant Adlakha, Mostafa Nouh, Chi Zhou, Peter W. Chung, Shenqiang Ren

**Affiliations:** 1 Department of Mechanical and Aerospace Engineering, University at Buffalo, The State University of New York, Buffalo, NY 14260 USA; 2 Weapons and Materials Research Directorate, US Army Combat Capabilities Development-Army Research Laboratory, Aberdeen Proving Ground, Aberdeen, MD 21005 USA; 3 Survice Engineering Co., Belcamp, MD 21017 USA; 4 Department of Mechanical Engineering, University of Maryland, College Park, MD 20740 USA; 5 Department of Materials Design and Innovation, University at Buffalo, The State University of New York, Buffalo, NY 14260 USA; 6 Department of Industrial and Systems Engineering, University at Buffalo, The State University of New York, Buffalo, NY 14260 USA; 7 Neutron Scattering Division, Oak Ridge National Laboratory, Oak Ridge, TN 37831 USA; 8 Research and Education in Energy Environment & Water Institute, University at Buffalo, The State University of New York, Buffalo, NY 14260 USA; 9 Department of Chemistry, University at Buffalo, The State University of New York, Buffalo, NY 14260 USA

**Keywords:** Devices for energy harvesting, Ferroelectrics and multiferroics

## Abstract

Chemical energy ferroelectrics are generally solid macromolecules showing spontaneous polarization and chemical bonding energy. These materials still suffer drawbacks, including the limited control of energy release rate, and thermal decomposition energy well below total chemical energy. To overcome these drawbacks, we report the integrated molecular ferroelectric and energetic material from machine learning-directed additive manufacturing coupled with the ice-templating assembly. The resultant aligned porous architecture shows a low density of 0.35 g cm^−3^, polarization-controlled energy release, and an anisotropic thermal conductivity ratio of 15. Thermal analysis suggests that the chlorine radicals react with macromolecules enabling a large exothermic enthalpy of reaction (6180 kJ kg^−1^). In addition, the estimated detonation velocity of molecular ferroelectrics can be tuned from 6.69 ± 0.21 to 7.79 ± 0.25 km s^−1^ by switching the polarization state. These results provide a pathway toward spatially programmed energetic ferroelectrics for controlled energy release rates.

## Abstract (web summary)

Chemical energy ferroelectrics show limited control of the energy release rate. Here, the authors design spatially programmed energetic ferroelectrics with controlled energy release rates from machine learning-directed additive manufacturing and ice-templating assembly.

## Introduction

**Fig. 1:**
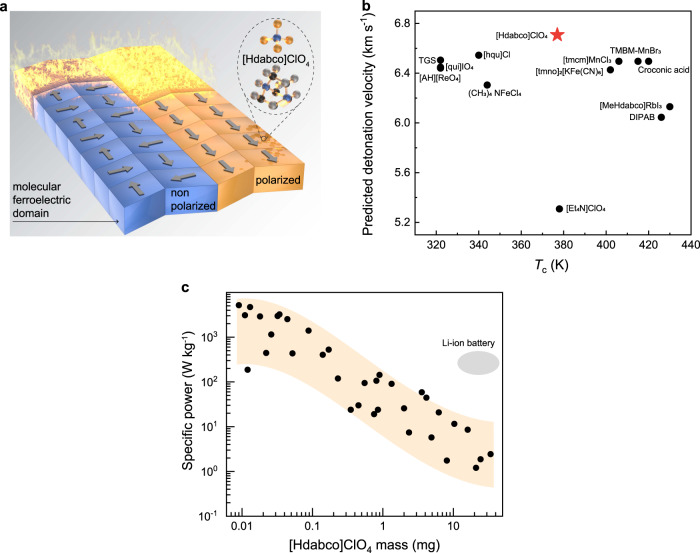
Energetic molecular ferroelectrics. **a** Schematic figure for the polarization control of decomposition for molecular ferroelectrics. The polarization induced ferroelectric domain structure change can cause the change in thermal conductivity, which further influence the detonation velocity. **b** Data-driven computational materials design. Results of *T*_c_ versus the predicted detonation for molecular ferroelectrics from machine learning models. **c** The mass dependent specific peak power for energetic [Hdabco]ClO_4_. The specific power is enhanced at the low-mass devices due to the larger surface aera.

Molecular energetic ferroelectrics, which store both chemical bond energy and undergo spontaneous polarization^1–3^, have stimulated interest in polarization-controlled energy release^4–6^, while self-assembly and additive manufacturing could further generate spatially programmed architecture of molecular energetic ferroelectrics with the aligned meso-structure promising for controlled energy flow (Fig. 1a)^5–8^. Such abilities offer the prospects of realizing molecular energetic ferroelectrics, a subset of chemical energy materials, with external stimuli dependent energy release^9,10^. While a large number of molecular ferroelectrics and energetics have been reported independently^11–15^, the exploration and structure engineering of energetic molecular ferroelectrics is still in infancy due to the lack of an effective material design approach^6^. We proposed the following design elements in order to create a material with a link between energetic and ferrielectric properties: (1) molecular ferroelectrics show the spontaneous polarization, high chemical-energy density, and estimated detonation velocity; (2) spatially programmed molecular ferroelectrics exhibit the aligned meso-structure for polarization-controlled energy release and energy flow. Following this design approach, we report machine learning-guided molecular energetic ferroelectrics with the capability of ice-templating assembly and additive manufacturing, for the control of spatially programmed architecture and aligned mesostructures.

The [Hdabco]ClO_4_ (dabco = 1,4-diazabicyclo[2.2.2]octane) is selected as the prototypical example of molecular energetic ferroelectric material for the study of polarization-directed detonation and heat of decomposition (Fig. 1b)^16,17^. The combination of ice-templating assembly and additive manufacturing enables to bridge across multiple length scale^18–21^, and the creation of three-dimensional (3D) aligned porous molecular energetic ferroelectrics with complex geometries, large surface area, and low density of 0.35 g cm^−3^. Pyrolysis-gas chromatography-mass spectrometry (GC/MS) and high-pressure differential scanning colorimetry (HP-DSC) analysis suggest that the Cl radicals from ClO_4_^−^ react exothermically with both cellulose and dabco, enabling a large exothermic heat of reaction (6180 kJ kg^−1^). Additionally, the aligned architecture produces an anisotropic thermal conductivity (ratio = 15) of 0.02 and 0.29 W mK^−1^ for the direction perpendicular and parallel to the aligned structure, respectively. The laser-induced air shock from energetic materials (LASEM) study shows that the polarization-dependent estimated detonation velocity can be tuned from 6.69 ± 0.21 to 7.79 ± 0.25 km s^−1^, generating a electricity with a specific power of 4.6 kW kg^−1^ (Fig. 1c) and controlled energy release rate.

## Results

For high-throughput screening of integrated molecular energetic ferroelectrics^8,22^, a two-step machine learning technique is applied. As shown in Supplementary Fig. 1, the first step is focused on predicting molecular ferroelectric candidates that can fulfill the design parameters mentioned above: water-soluble characteristics for ice-templating assembly, high polarization, and Curie temperature (*T*_c_). The second step predicts the detonation velocity based on the Kernel Ridge Regression and E-state Fingerprint model (Supplementary Tables S1) for further down-selecting candidates with high chemical-energy density and high energy release rate^22^. Figure 1b and Supplementary Tables S2–S3 show the predicted detonation velocities for some representative water-soluble molecular energetic ferroelectrics obtained from machine learning. The aqueous processable [Hdabco]ClO_4_ is selected for the following study due to its high predicted detonation velocity, a *T*_c_ above room-temperature, and large pyroelectric coefficient for thermal energy conversion (Supplementary Fig. 2)^23^.

### Structural and dielectric properties of energetic [Hdabco]ClO_4_

**Fig. 2:**
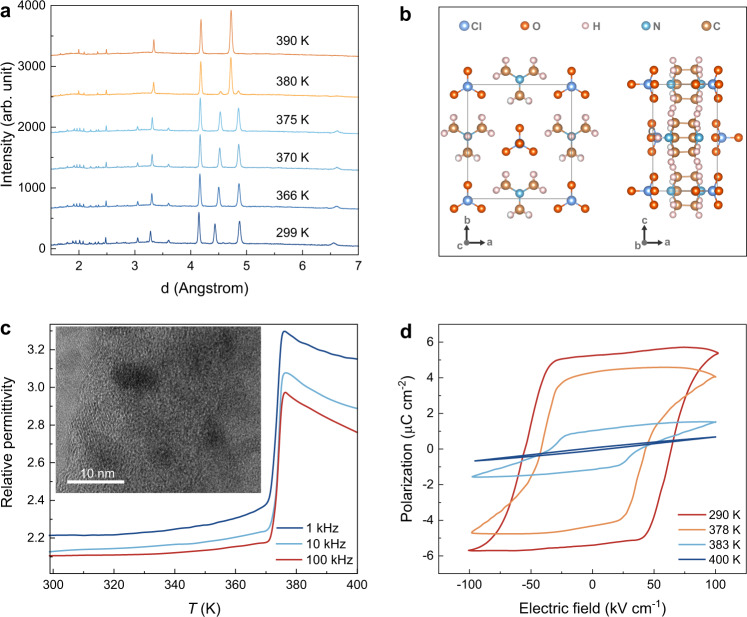
Structural and dielectric properties of energetic [Hdabco]ClO_4_. **a** Neutron diffraction patterns at different temperature for energetic [Hdabco]ClO_4_. **b** Crystal structure for the ferroelectric phase of energetic [Hdabco]ClO_4_. **c** Temperature dependence of relative permittivity for energetic [Hdabco]ClO_4_ at 1, 10, and 100 kHz. Inset is the HRTEM image for energetic [Hdabco]ClO_4_ showing distinct fringes. **d** Polarization-electric field loops for energetic [Hdabco]ClO_4_ at different temperatures.

Neutron diffraction measurements at different temperatures are performed on [Hdabco]ClO_4_ (Fig. 2a, Supplementary Figs. 3–8 and Supplementary Table S4–S16). As shown in Fig. 2b and Supplementary Table S5, [Hdabco]ClO_4_ adopts the noncentrosymmetry *Pm*2_1_*n* space group with lattice parameters of *a* = 8.8716 Å, *b* = 9.7501 Å, and *c* = 5.3534 Å at 298 K. The high-resolution transmission electron micrograph (HRTEM) shows that the sample exhibits significant and uniform crystallinity with pronounced Moire fringes (inset of Fig. 2c and Supplementary Figs. 9–11). As the temperature increases, a first-order phase transition to a high-temperature centrosymmetric phase occurs. Neutron diffraction shows the transition happens between 375 and 390 K. A coexistence of low-temperature and high-temperature phase is observed at 380 K, while high-temperature phase adopts a *P*4*/mmm* space group with the lattice parameters of *a* = *b* = 9.4512 Å and *c* = 5.3710 Å at 390 K (Supplementary Figs. 8 and 12, and Supplementary Table S16). Temperature dependence of dielectric measurement shows a dielectric anomaly at the phase transition temperature of 377 K (Fig. 2c). The ferroelectricity is also supported by the polarization–electric field (*P*–*E*) loops and current–electric field (*I*–*E*) curve (Fig. 2d and Supplementary Fig. 13). The [Hdabco]ClO_4_ shows coercivity of 54 and 64 kV cm^−1^ for negative and positive electric field, respectively. The difference of 10 kV cm^−1^ in coercivity originates from the self-poling field formed during the growth of [Hdabco]ClO_4_^16,23^.

### Structure design of energetic [Hdabco]ClO_4_ by additive manufacturing coupled with ice-templating

**Fig. 3:**
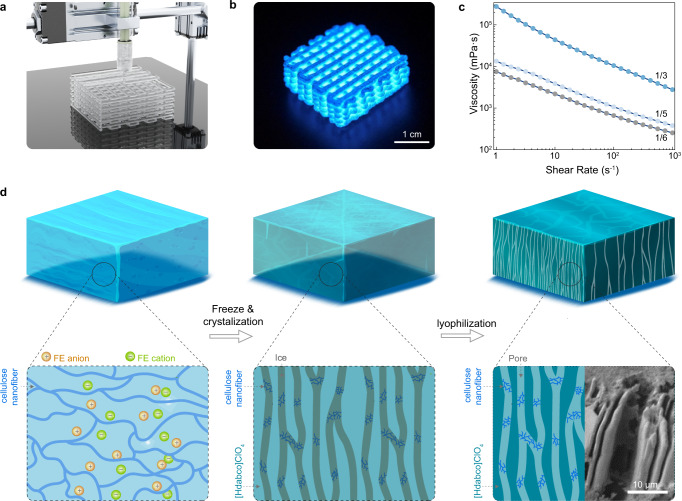
Structure design of energetic [Hdabco]ClO_4_ by additive manufacturing coupled with ice-templating. **a** The schematic diagram of an extrusion-based 3D printing. **b** Optical image for the 3D printed [Hdabco]ClO_4_. Fluorescent colorants are added for imaging. **c** Shear-thinning behavior of precursor with different weight ratio (cellulose/[Hdabco]ClO_4_). **d** Schematics of freeze-drying process. (Inset) SEM image for the ice-templated [Hdabco]ClO_4_.

Additive manufacturing (3D printing) enables the rapid prototyping of energetic materials through the layer-by-layer deposition, providing the pathway towards the unprecedented geometry control over thermal decompaction behavior^24^. The 3D printing coupled with ice-templating assembly produces the aligned porous [Hdabco]ClO_4_ microstructure promising for the effective energy flow and thermal decomposition. As shown in Fig. 3a and Supplementary Movie 1, energetic [Hdabco]ClO_4_ is printed by direct ink writing from precursors consisting of the saturated [Hdabco]ClO_4_ solution and cellulose nanofiber mixture at different weight ratios. Figure 3b shows the optical image of the 3D printed energetic [Hdabco]ClO_4_. Cellulose nanofiber is chosen as the additive to enhance 3D printability and effective thermal decomposition for molecular energetic ferroelectrics. As shown in Fig. 3c, the ink exhibits the shear thinning behavior required for direct ink writing. The viscosity of the ink increases as the weight ratio of cellulose increases. Figure 3d shows the ice-templating assembly integrated crystallization process from 3D printing. Before freezing, the cellulose and dissolved [Hdabco]ClO_4_ are homogeneously distributed in the printable precursor slurry (Supplementary Fig. 14). After placing the printed parts at 253.15 K for unidirectional freezing, water molecules form ice while [Hdabco]^+^ cations and ClO_4_^−^ anions crystallize simultaneously. The ice and [Hdabco]ClO_4_ crystals expel each other while the cellulose is mainly located inside the ice crystals. After freeze drying, a porous structure with the aligned [Hdabco]ClO_4_ crystal mesostructured is obtained. The unidirectional freezing induces the temperature gradient for the formation of microscale crystallites oriented preferentially along the direction of freezing, leading to the resulting scaffolds with the lamellar [Hdabco]ClO_4_ structures. Density-functional theory (DFT) and control experiments on single crystal growth shows that the head-to-tail one dimensional hydrogen bond between the adjacent [Hdabco]^+^ cations induces self-assembly of [Hdabco]ClO_4_ (Supplementary Figs. 15–17). Furthermore, the hydrogen bonding between cellulose and [Hdabco]ClO_4_ facilitates the formation of porous architecture in the 3D printing process.

### Structural properties and thermal analysis of 3D printed [Hdabco]ClO_4_

**Fig. 4:**
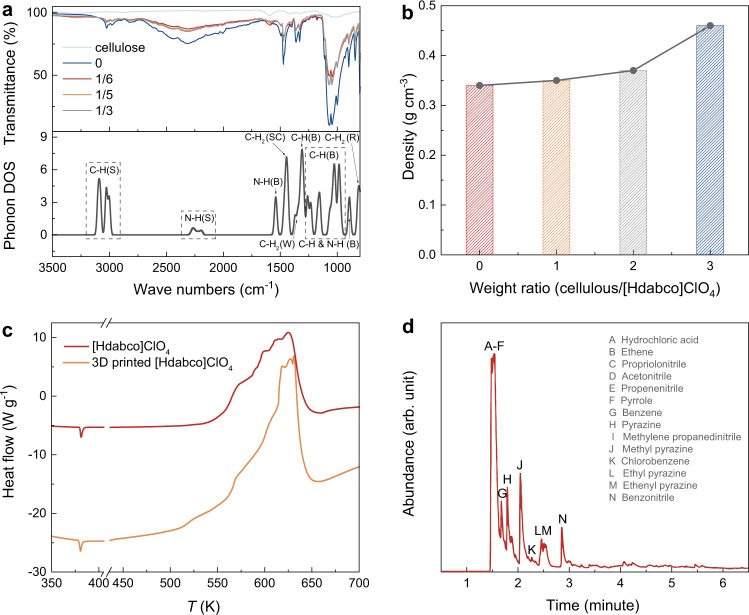
Structural properties and thermal analysis of 3D printed [Hdabco]ClO_4_. **a** FTIR spectra for cellulose and printed sample different weight ratio (cellulose/[Hdabco]ClO_4_). Calculated phonon density of states (DOS) for [Hdabco]ClO_4_. bending (B); stretching (S); rocking (R); wagging (W); scissoring (SC). The phonon dispersion is shown in Supplementary Fig. 18. **b** Weight ratio (cellulose/[Hdabco]ClO_4_) dependent density for printed energetic [Hdabco]ClO_4_. **c** HP-DSC traces for [Hdabco]ClO_4_ and 3D printed [Hdabco]ClO_4_ (weight ratio of cellulose/[Hdabco]ClO_4_ = 1/5). **d** Total ion chromatogram for pyrolysis of 3D printed [Hdabco]ClO_4_ (weight ratio of cellulose/[Hdabco]ClO_4_ = 1/5) at 400 °C.

The Fourier transform infrared (FTIR) spectroscopy measurements are performed on the cellulose, [Hdabco]ClO_4_, and 3D aligned porous architecture samples with different weight ratios (Fig. 4a). The phonon density of states (DOS) is calculated for [Hdabco]ClO_4_ (Fig. 4a). The FTIR peaks for [Hdabco]ClO_4_ doesn’t show obvious change after 3D printing process, showing the stability of 3D printed samples. The ice-templating coupled with additive manufacturing enables a network of linked cellulose and high porosity in the printed [Hdabco]ClO_4_ (Supplementary Figs. 19–21), where its tunable densities of 0.34, 0.35, 0.37, and 0.46 g cm^−3^ are obtained at different weight ratios (cellulose/[Hdabco]ClO_4_) of 0, 1/6, 1/5, and 1/3, respectively (Fig. 4b). Increasing the cellulose would increase the density and reduce the pores evidenced by the scanning electron microscopy images (SEM, Supplementary Fig. 21). Supplementary Fig. 22 show the N_2_ adsorption-desorption isotherms and the pore size distribution of 3D printed [Hdabco]ClO_4_ (weight ratio = 1/5). The N_2_ sorption isotherms (type IV) show an H3 hysteresis loop, indicating that the product has a typical mesoporous structure^25^. The Brunauer-Emmett-Teller surface area analyses show that the specific surface areas of printed [Hdabco]ClO_4_ are 1.4125, 1.3859, and 1.1036 m^2^ g^−1^ for the weight ratio (cellulose/[Hdabco]ClO_4_) of 1/6, 1/5, and 1/3, respectively^26^. The explosive nature of molecular energetic ferroelectrics generally leads to low heat of decomposition due to its volatilization. We further study chemical decomposition to explore the heat of decomposition properties in the 3D printed [Hdabco]ClO_4_. HP-DSC for [Hdabco]ClO_4_ under N_2_ shows that enthalpy of decomposition increases with increasing N_2_ pressure (Supplementary Figs. 23 and 24). A large enthalpy of decomposition of 4898 kJ kg^−1^ at 200 psi, enabled by the suppression of [Hdabco]ClO_4_ volatilization under pressure. The decomposition of 3D printed [Hdabco]ClO_4_ is also studied under 200 psi N_2_. Figure 4c shows enthalpy of decomposition for 3D printed [Hdabco]ClO_4_ is 5922 kJ kg^−1^, which exceeds the calculated expected value (4511 kJ kg^−1^) according to the weight ratio of cellulose and [Hdabco]ClO_4_ in the printed sample. GC/MS analysis (Fig. 4d) for the desorption (D) and pyrolysis (P) products of 3D printed [Hdabco]ClO_4_ shows that chlorine is, for the most part, absent in the D/P products of 3D printed [Hdabco]ClO_4_. Given that high reactivity of chlorine radicals and highly exothermic chlorination of organic materials, it is reasonable to assume that a large fraction of Cl radicals from ClO_4_^−^ react with both cellulose and dabco (Supplementary Figs. 25–47). The exothermic reaction of Cl radicals gives a large exothermic heat of reaction of 6180 kJ kg^−1^ (Supplementary Figs. 48).

### Ferroelectricity control of chemical energy release

**Fig. 5:**
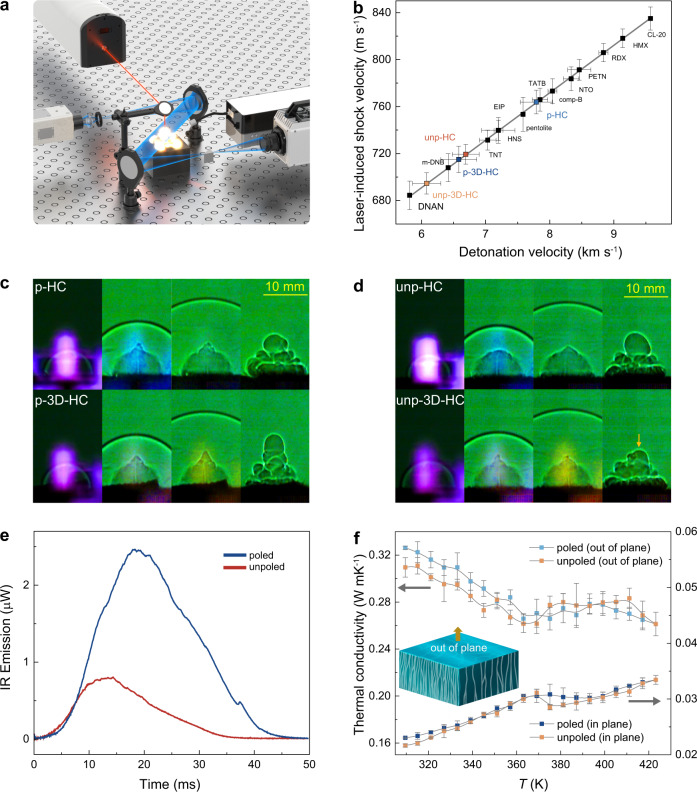
Ferroelectricity control of chemical energy release. **a** Schematic figure for laser-induced air shock from energetic materials study. The light from arc lamp is focused through a slit onto the first mirror. The change in the refractive index of air above the sample surface is detected by collimated light between the first and second mirrors. A knife edge is used to cut about half of the light rays. A spectrometer is used to measure the plasma emission spectrum at the same time and an IR photoreceiver records the time-resolved combustion emission. **b** The laser-induced shock and estimated detonation velocities of poled, unpoled, 3D printed poled, and 3D printed unpoled [Hdabco]ClO_4_. Error bars represent 95% confidence intervals. **c** Selected high-speed-video snapshots from for laser-shocked poled and 3d printed poled [Hdabco]ClO_4_, starting at time = 0 μs for the first frame with each subsequent frame 11.9 μs later. **d** Selected high-speed-video snapshots from for laser-shocked unpoled and 3d printed unpoled [Hdabco]ClO_4_. **e** IR emission for 3D printed [Hdabco]ClO_4_ (weight ratio = 1/5) measured during the LASEM experiments. **f** Temperature dependence of thermal conductivity for poled and unpoled [Hdabco]ClO_4_.

We further explore the ferroelectric polarization control of chemical energy release. The energy release of energetic [Hdabco]ClO_4_ is studied by the LASEM method^27^. In the measurement, the energetic [Hdabco]ClO_4_ is ablated into a high-temperature plasma and then high-speed schlieren images of the laser-induced shock wave are obtained to measure the microsecond-timescale energy release (Fig. 5a). Four samples are measured, including poled [Hdabco]ClO_4_ (p-HC), unpoled [Hdabco]ClO_4_ (unp-HC), poled 3D printed [Hdabco]ClO_4_ (p-3D-HC), and unpoled 3D printed [Hdabco]ClO_4_ (unp-3D-HC). As shown in Fig. 5b, the obtained laser-induced shock velocities are 763.86 ± 10.06, 719.32 ± 8.35, 714.97 ± 11.22, and 694.56 ± 9.00 m s^−1^ for p-HC, unp-HC, p-3D-HC and unp-3D-HC, respectively. The estimated detonation velocities are 7.79 ± 0.25, 6.69 ± 0.21, 6.58 ± 0.28, and 6.08 ± 0.22 km s^−1^ for p-HC, unp-HC, p-3D-HC and unp-3D-HC, respectively. Representative snapshots from the poled (Fig. 5c) and unpoled (Fig. 5d) samples demonstrate the formation of the laser-induced plasma (first frame), the expansion of the laser-induced shock wave into the air above the sample, the combustion of cellulose (3^rd^ frame of 3D-HC samples), and the decrease in combustion gases produced from unp-3D-HC compared to p-3D-HC (last frame). Compared with the p-HC, the p-3D-HC shows reduced energetic performance since it only contains 83% p-HC. Cellulose has a high heat of decomposition resulting from combustion, but reactions with air are slow-they do not affect the detonation velocity. After depolarizing [Hdabco]ClO_4_, the laser-induced shock velocity is reduced by 5.8% and the estimated detonation velocity decreased by 14%, evidencing the polarization control of energetic performance. For the 3D printed sample, removing polarization reduces the estimated detonation velocity by 7.7%; the effect is reduced compared to neat [Hdabco]ClO_4_ because of the smaller concentration of p-HC in the material. The integrated plasma emission spectra (1.5 μs delay with a 10 μs gate width) and time-resolved infrared (IR) emission are also measured during the LASEM experiments. After the 3D printed sample is depolarized, the C and Cl plasma emissions remain unchanged, while the Na and H emissions increase (Supplementary Fig. 49). This is in contrast to the unp-HC, which shows a decrease in C emission and an increase in Cl and H emissions when compared with p-HC (Supplementary Fig. 50). This suggests that the presence of cellulose in the 3D-printed sample influences the decomposition mechanism of p-HC. The IR emission enables comparison of the combustion reactions with air on the millisecond timescale. The presence of cellulose increases the intensity and duration of the combustion emission for p-3D-HC compared to p-HC and depolarization significantly reduces the combustion reactions in both cases (Fig. 5e and Supplementary Fig. 51).

### The control of chemical energy release

**Fig. 6:**
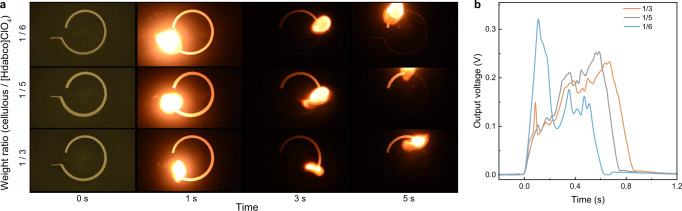
The control of chemical energy release. **a**, Selected snapshots from the video for the decomposition of 3D printed [Hdabco]ClO_4_ with different weight ratios of cellulose/[Hdabco]ClO_4_. **b** The decomposition generated electricity from [Hdabco]ClO_4_ with different weight ratios of cellulose/[Hdabco]ClO_4_.

To further understand the polarization control of energetic performance in [Hdabco]ClO_4_, the temperature dependence of thermal conductivity for the polarized and unpolarized [Hdabco]ClO_4_ are measured. The effective thermal conductivity can be tuned by controlling the ferroelectric domain structure and polarization^28–30^. The aligned crystalline structure of [Hdabco]ClO_4_ shows anisotropic thermal conductivities for the parallel and perpendicular directions (Fig. 5f), which results in anisotropic thermal conduction based on the thermal response simulation (Supplementary Fig. 52). During the heating process, thermal conductivity shows a turning point when approaching *T*_c_ due to the ferroelectric-to-paraelectric phase transition. For the poled state at 309 K, thermal conductivities are 0.023 ± 0.0001 and 0.326 ± 0.0014 W mK^−1^ for perpendicular and parallel direction to the aligned structure, respectively. Compared with polarized samples, the unpolarized samples show low thermal conductivity for both directions at 309 K, which is mainly attributed to the dipole-induced change of phonon-phonon scattering and ferroelectric domain structures^16^. Such polarization dependence of thermal conductivity is promising for the control of chemical energy release. In addition, the decomposition rate could also be controlled by varying the weight ratios between cellulose and [Hdabco]ClO_4_ components. As shown in Fig. 6a and Supplementary Movie 2, when the ratio increases from 1/6 to 1/3, the decomposition rate is reduced, leading to the control of pyroelectric mediated conversion from chemical to electrical energy. A controlled reaction rate from 0.63 to 0.87 s is obtained by changing the weight ratios (Fig. 6b).

In summary, energetic molecular ferroelectric [Hdabco]ClO_4,_ derived from machine learning guided ice-templating and additive manufacturing, shows a polarization-controlled detonation velocity and high heat of decomposition. The GC/MS and HP-DSC analyses suggest that Cl radicals react with macromolecules enabling a large exothermic heat of reaction (6180 kJ kg^−1^). Under a poled state at 309 K, the 3D printed lightweight porous energetic [Hdabco]ClO_4_ with the aligned mesostructured shows an anisotropic thermal conductivity of 0.023 ± 0.0001 and 0.326 ± 0.0014 W mK^−1^ for perpendicular and parallel direction to the aligned structure, respectively. The LASEM study shows that the estimated detonation velocity can be tuned from 6.69 ± 0.21 to 7.79 ± 0.25 km s^−1^ by the polarization dependence of thermal conductivity. The large heat of decomposition and tunable energy release rate enables a controllable chemically driven electricity with a large specific power of 4.6 kW kg^−1^.

## Methods

### Synthesis of [Hdabco]ClO_4_

[Hdabco]ClO_4_ crystals are synthesized by slow evaporation of the aqueous solution of 1,4-Diazabicyclooctane triethylenediamine (Sigma-Aldrich) and perchloric acid (70%, Sigma-Aldrich) in a 1:1 molar ratio.

### 3D printing and ice-templating process

The printable ink is prepared by adding cellulose nanofiber (Nanografi Nano Technology) to the saturated [Hdabco]ClO_4_ solution at various ratios. The ink is loaded into a 5 mL syringe barrel (Nordson EFD, USA), connected with an 800μm nozzle (Nordson EFD, USA). The ink is extruded through an air pressure (3.0 psi) powered dispenser (Nordson EFD, USA) for the direct ink writing process. The dispenser is mounted onto a retrofitted printer (Ultimaker, Netherlands). G-code files are generated with open-source software Slic3r and are used to direct the XYZ motion of the printing system to build 3D architectures. After printing, samples are transferred to a freezer (253 K) and placed one a cold aluminum plate for 2 h. The frozen samples are freeze-dried using a freeze-drying system (LABCONCO, USA) operating under 0.015 mBar vacuum. The primary drying process starts from 233 K then ramped to 263 K at a rate of 0.2 K min^−1^ and holds at −10 °C for 10 h. Next, the secondary drying process ramps to 278 K at a rate of 0.5 K min^−1^ and holds for 6 h. Finally, the temperature is elevated to 293 K and held for 2 h to allow completely solvent sublimation.

### Structure and elemental analysis

Hitachi S4000 SEM microscope is used for obtaining the SEM and EDS images. For FTIR measurements, an Agilent Cary 630 FTIR spectrometer (Agilent Technologies, Inc., USA) is used.

### Thermal analysis

Thermal analysis in the air is performed by a SDT Q600 Simultaneous Differential Scanning Calorimeter/Thermogravimetric Analyzer (TA Instruments, USA) at 10 K min^−1^. The thermal conductivity is obtained from a Hot Disk TPS 2200 instrument (Hot Disk AB, Sweden). High-pressure DSC analyses were carried out using a TA Instruments Q20 DSC at pressures up to 450 psi. All samples were analyzed at a heating rate of 10 K min^−1^ under a nitrogen flow (50 mL/min). Samples were held in crimped “standard” aluminum pans (pans: TA instrument PN 900786.901; lids: TA Instruments PN 900779.901). No evidence of oxidation of the pans was observed.

### Pyrolysis-gas chromatography-mass spectrometry (GC/MS)

Products from desorption and pyrolysis are examined using a GC/MS analyzer^6^. An Agilent (Santa Clara, California) GC/MS system’s splitless injector is coupled to a CDS Analytical Model 2000 Pyroprobe (coil type) for desorption (Model 6890 N GC and Model 5973 N MSD). A HP-5 capillary column is utilized for the GC column. The Pyroprobe interface and injector temperature is 250 °C. Pyroprobe is configured to provide a 20 s desorption pulse by heating from 175 to 400 °C at 1000 °C/s. The analyses are performed on a single sample. Sample is kept in the Pyroprobe’s coil and weighed around 1 mg.

### Dielectric and electrical characterization

*P*–*E* hysteresis loops and *I*–*E* curve are obtained with a Precision LC ferroelectric analyzer (Radiant Technologies Inc., USA). Temperature dependence of the dielectric constant is measured with an 4294 A impedance analyzer (Agilent Technologies, Inc., USA). The temperature environment is controlled by the Physical Properties Measurement System EverCool-II^TM^ (Quantum Design, Inc., USA). A Keithley 2450 SourceMeter SMU instrument (Tektronix, USA) is used to measure the chemically driven electricity. Samples are polarized by a saturated electric field (100 kV cm^−1^).

### LASEM measurements

The LASEM system is previously described in detail^27^; briefly, samples are prepared by pressing a thin layer of material (~10 mg) on double-sided tape affixed to a glass slide. A 6 ns pulsed laser (1064 nm, 850 mJ, 180 J cm^−2^) is used to ablate, atomize, ionize, and excite the samples. The chemical reactions in the resulting laser-induced plasma mimic the chemistry behind the detonation front of an explosion and influence the expansion of the laser-induced shock wave into the air. The laser-induced shock velocities are measured using high-speed schlieren imaging (84 kfps; 1 μs shutter) and can be used to estimate the detonation velocity of the material, assuming the material is detonable and the chemistry is similar to that of conventional military explosives. The emission spectra from the laser-induced plasma were obtained with a high-resolution echelle spectrometer equipped with an intensified charge coupled device detector (Catalina Scientific SE200 with Apogee detector; gate delay = 1.5 μs, gate width = 10 μs, 200–1000 nm, λ/Δλ = 2700). The integrated emission from the combusting particles on the millisecond timescale was monitored with an IR-sensitive photoreceiver (New Focus model 2053; 900–1700 nm). Data from 20 laser shots were acquired from each sample.

### TEM analyses

All TEM experiments are performed using a JEOL JEM 2100 F TEM located at the US Army Combat Capabilities Development Command – Army Research Laboratory (DEVCOM ARL) operated at 200 keV accelerating voltage. The TEM specimens are prepared by suspending [Hdabco]ClO_4_ colloidal solutions in acetone onto the holey carbon support film of TEM copper grids (300 mesh, Ted Pella, Inc.). High resolution bright field images are acquired in the TEM mode while high-angle annular dark-field (HAADF) images are acquired to obtain multi-element mapping and chemical composition quantification using a Tridiem Gatan Image Filter (GIF) and the EDAX EDS detector in scanning TEM mode through the TEAM Analysis software (EDAX, Inc).

### Power neutron diffraction experiments

Power neutron diffraction experiments are conducted at the time-of-flight (TOF) powder diffractometer (POWGEN), located at the Spallation Neutron Source at Oak Ridge National Laboratory. A powder sample of ~1.6 g is loaded in an 8 mm diameter vanadium PAC can. A vacuum furnace is adopted as the sample environment to cover high temperature region. High resolution neutron diffraction patterns are collected at a couple of temperatures between 300 and 390 K using the neutron frame with a center wavelength of 1.5 \AA. The Rietveld analysis on the data is performed using the FullProf refinement suite.

### DFT calculation

The density functional theory (DFT) calculations were performed using the QUANTUM ESPRESSO^31^ package with the projector-augmented wave (PAW) method^32^. The generalized gradient approximation (GGA) of the Perdew-Burke-Ernzerhof (PBE)^33^ form of the exchange-correlation functional was used with a plane wave energy cutoff of 74 Ry. The Grimme-D2^34^ dispersion correction was applied to account for the Van der Waals (vdW) interactions. The Brillouin zone was sampled with a $4\times 4\times 6$ mesh for structural optimization and self-consistent calculations. The force and energy convergence criteria were set to $1\times {10}^{-6}$Ry/Bohr $1\times {10}^{-7}$ Ry, respectively. The second-order force constants were calculated using the density functional perturbation theory (DFPT)^1^ with a $3\times 3\times 4$ Monkhorst-Pack q point mesh. A $20\times 20\times 20$ q grid was used to calculate the phonon density of states.

### Machine Learning

#### Featurization and property predictor

Five different featurization methods are used in this paper: Sum Over Bonds (SoB), E State, a Custom Descriptor Set (CDS), Joint Embedding^35^, and a concatenation of Sum Over Bonds, E State and Customer Descriptor Set^22^. The set of features was shown earlier to result in less mean absolute error (MAE) and improved performance for the estimation of exothermic reaction parameters^35,36^.

Eight separately trained machine learning models are examined as property predictors, that takes an input feature and predict detonation velocity as the output. The eight models include Gaussian Process Regression, Kernel Ridge Regression, Support Vector Regression, Random Forest, Lasso Regression, k-Nearest Neighbors, Gradient Boosting and Ridge Regression. Previous studies on structure property prediction in energetic materials have shown good performance with these models^35,36^. Hyperparameter optimization is performed on each model (and for each featurization) using grid search with 5-fold cross validation. Careful hyperparameter optimization is necessary while evaluating different models.

#### Dataset

Energetic materials used in this study comes from Huang and Massa^37^ and Mathieu^36^ containing 109 and 307 compounds respectively. The ferroelectric materials^8^ come from of which the detonation velocity for three ferroelectric materials are known^6,38,39^. We use three random train/test splits of 2/1 from among the smaller set of three ferroelectric materials with known detonation velocity values, while keeping the entire set of energetic materials to train the machine learning models. This means that in each instance in training the property predictor, the data are comprised of 401 energetic molecules and 2 molecules from the known ferroelectric set. MAE is averaged over the three random train test splits, and the best model and featurization scheme that gives the minimum MAE is chosen. Autoencoder in the joint embedding framework is trained with 100 K molecules from MOSES^40^ in addition to the entire set of 401 energetic molecules^35^. Finally, the best model and featurization scheme chosen from model cross validation process is used to predict the detonation velocity of each ferroelectric material in the dataset.

### Reporting summary

Further information on research design is available in the Nature Research Reporting Summary linked to this article.

## Supplementary information

Supplementary Information

Description of Additional Supplementary Files

Supplementary Movie 1

Supplementary Movie 2

Lasing Reporting Summary

## Data Availability

The data generated in this study are provided within the manuscript and Supplementary Information file. Any additional information needed is available from the corresponding author upon request.
