# Supplementary material for: Releasing chemical energy in spatially programmed ferroelectrics
Source: Nat Commun. 2022 Nov 15;13:6959. doi: 10.1038/s41467-022-34819-z (PMC9666659; doi:10.1038/s41467-022-34819-z)
Supplement: Supplementary file 1 — Supplementary Information [file 41467_2022_34819_MOESM1_ESM.pdf]

Supplementary Information for

Releasing Chemical Energy in Spatially Programmed Ferroelectrics

Yong Hu[1], Jennifer L. Gottfried[2], Rose Pesce-Rodriguez[2], Chi-Chin Wu[2], Scott D. Walck[3], Zhiyu Liu[4], Sangeeth Balakrishnan[4], Scott Broderick[5], Zipeng Guo[6], Qiang Zhang[7], Lu An[1], Revant Adlakha[1], Mostafa Nouh[1], Chi Zhou[6], Peter W Chung[4,] and Shenqiang Ren[1,8,9*]

Affiliations:

[1]Department of Mechanical and Aerospace Engineering, University at Buffalo, The State University of New York, Buffalo, NY 14260, USA

[2]Weapons and Materials Research Directorate, US Army Combat Capabilities Development-Army Research Laboratory, Aberdeen Proving Ground, Aberdeen, MD 21005, USA

[3]Survice Engineering Co., Belcamp, MD 21017, USA

[4]Department of Mechanical Engineering, University of Maryland, College Park, MD 20740, USA

[5]Department of Materials Design and Innovation, University at Buffalo, The State University of New York, Buffalo, NY 14260, USA

[6]Department of Industrial and Systems Engineering, University at Buffalo, The State University of New York, Buffalo, NY 14260, USA

[7]Neutron Scattering Division, Oak Ridge National Laboratory, Oak Ridge, TN 37831, USA

[8]Research and Education in Energy Environment & Water Institute, University at Buffalo, The State University of New York, Buffalo, NY 14260, USA

[9]Department of Chemistry, University at Buffalo, The State University of New York, Buffalo, NY 14260, USA

*Correspondence to: shenren@buffalo.edu

Machine learning procedure for predicting water-soluble molecular ferroelectrics and a high T_c

The machine learning based modeling of T_c and water solubility incorporated multiple steps: the development of an appropriate feature set, the conversion of the data to an alternate format through manifold learning which maximizes the information while minimizing noise and error in the data, and the application of a quantitative prediction on the dimensionally reduced space.

Development of Feature Space: The features were first developed through the utilization of prior existing approaches[1,2]. The descriptors are largely based on the scaling from composition, where the initial feature set largely contains data existing in the periodic table, and therefore data which is available to predict unknown systems of interest. Additionally, bonding and stereochemistry are accounted for in the developed feature space and the data, therefore, provides a complete, complex and multi-scale design space. The feature space was incorporated with the property measurements for T_c and water solubility. The same initial feature space was used for both properties, and the dimensionality reduction followed was the same for both properties, as the underlying mathematics identified and addressed the unique relationships across property and feature. The difference in the two properties was then in the final development of the regression model.

*Development of Parameterization of Feature Space*Note, the entire feature set was used in building the dimensionality reduction; however, the properties were not as that would effectively introduce the answer into the analysis. Following the dimensionality reduction, the data was divided into a 80/20 training/test data split. The process was repeated five times to ensure that the results were not sensitive to the random selection of training and test data. The prediction was then developed and if the test RMSE was within 10% of the training RMSE, it was determined that the model was sufficiently robust. The logic of including all systems though in the dimensionality reduction step is that since the features are defined so as to only incorporate descriptors for all systems, the input does not require any special analysis or data measurement prior to analysis. By defining the analysis in this way, the robustness and transferability are maximized.

The parameterization of the data was done following the IsoMap approach for non-linear dimensionality reduction, or as used in this case for parameterization of complex data. This approach generates a graph connecting data points on a high dimensional space to their nearest neighbors, mapped out in the high dimensional space, and then fit a low dimensional manifold[2-5].The Isomap algorithm maps the distribution of elements in the high dimensional space, represented by the set of data points $\{x_i\} \in R^n$, onto a convex nonlinear manifold M^d of lower dimension $d < n$ and through dimensionality reduction, obtain a two or three dimensional embedding of the elements into a weighted graph. The mapping is carried out such that the geodesic distances between the elements in the higher dimensional manifold are preserved when it is mapped onto the lower-dimensional graph.

In order to construct the initial graph in R^n, we used K nearest-neighbors (KNN), which graphs each point connected by an edge to its 'k' nearest neighbors. The choice of k was optimized by statistically determining the smallest value that could minimize the residual variance $| d_M - d_G |$, while providing the maximum number of alternative paths. This ensures that the resulting graph is neither over-connected, leading to loss of pairwise geodesic distances, nor are critical neighbors disconnected.

Development of Robust High-Throughput Model: The IsoMap analysis provides a new parameterization, which serves as a more efficient representation of the data, while reducing dimensionality and the risk of over-fitting, as well as reducing noise and data sensitivity in the analysis. The regression approach employed was partial least squares (PLS)[2-7]. In PLS the training data is converted to a data matrix with orthogonalized axes, which are based on capturing the maximum amount of information in fewer dimensions. The relationships discovered in the training data can be applied to a test dataset based on a projection of the data onto a high-dimensional hyperplane within the orthogonalized axis-system. With PLS, the properties of the composites can be modeled as a function of the chemical and additive descriptors independent of each other. Typical linear regression models do not properly account for the co-linearity between the descriptors, and as a result, the isolated impact of each descriptor on the property cannot be accurately known. However, by projecting the data onto a high-dimensional space defined by axes which are comprised of a linear combination of the composite descriptors and also orthogonalized, the impact of the descriptor on the property can be identified independent of all other descriptors. This analysis, therefore, provides a unique approach that incorporates multiple steps, including the development of a feature set providing a multi-scale of information, the parameterization of the data through a non-linear manifold learning approach, and the development of a high-throughput regression linking the parameters and the property space. This approach is applied here to a specific problem, but it is anticipated to be general for a variety of relevant design objectives.

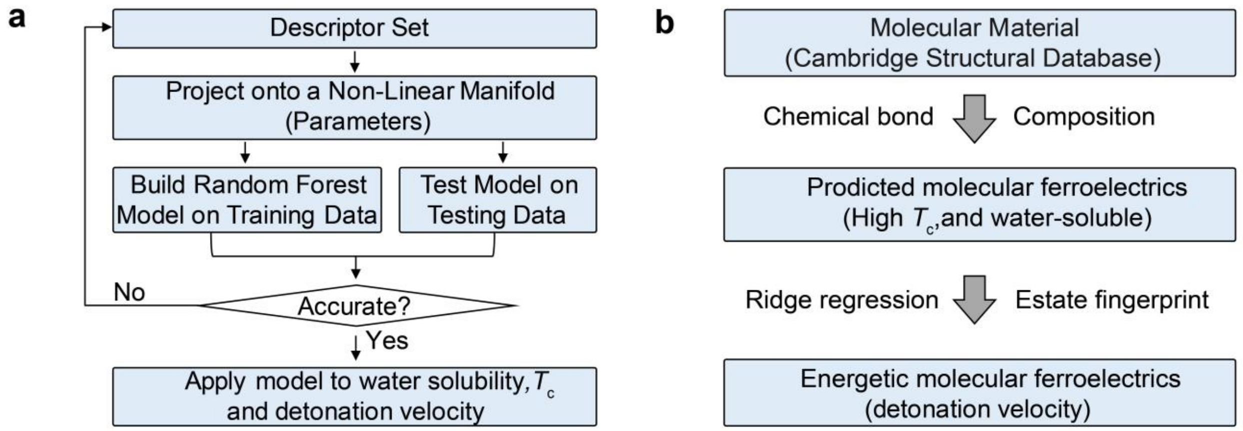

Supplementary Fig. 1. Data-driven computational materials design at the scale of microstructure enabled by supervised machine learning.

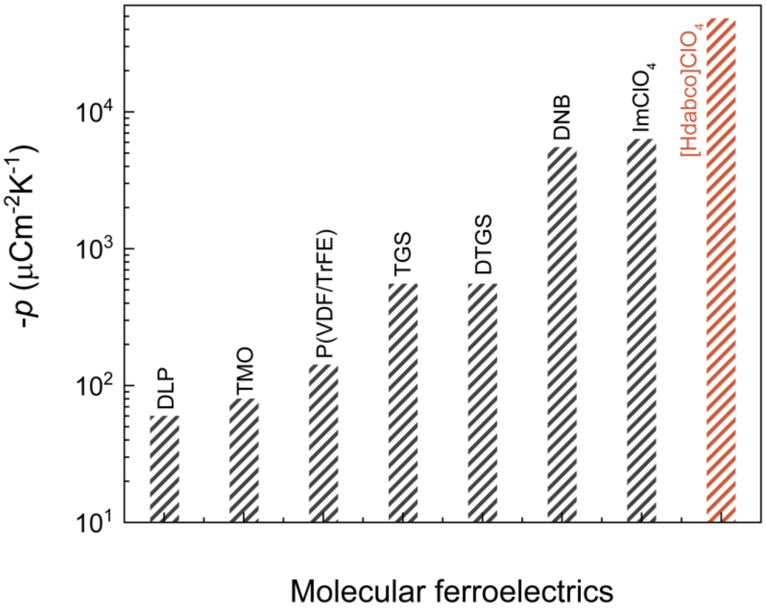

Supplementary Fig. 2. Pyroelectric coefficients (p) for representative molecular ferroelectrics with [Hdabco]ClO_4 highlighted in red.

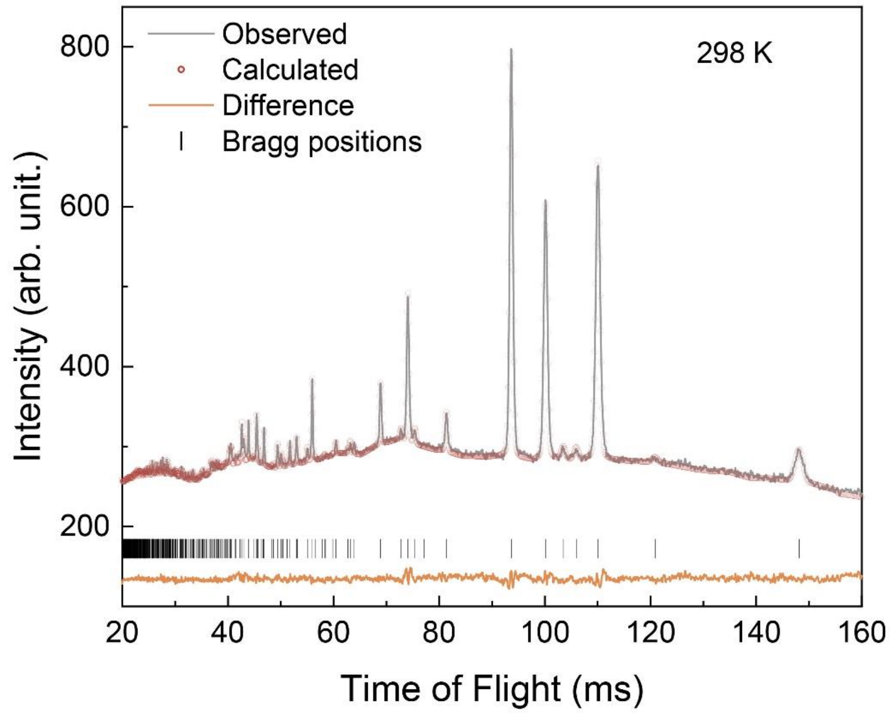

Supplementary Fig. 3. Rietveld refinement for the time-of-flight neutron diffraction profiles measured for $[Hdabco]ClO_4$ at 298 K.

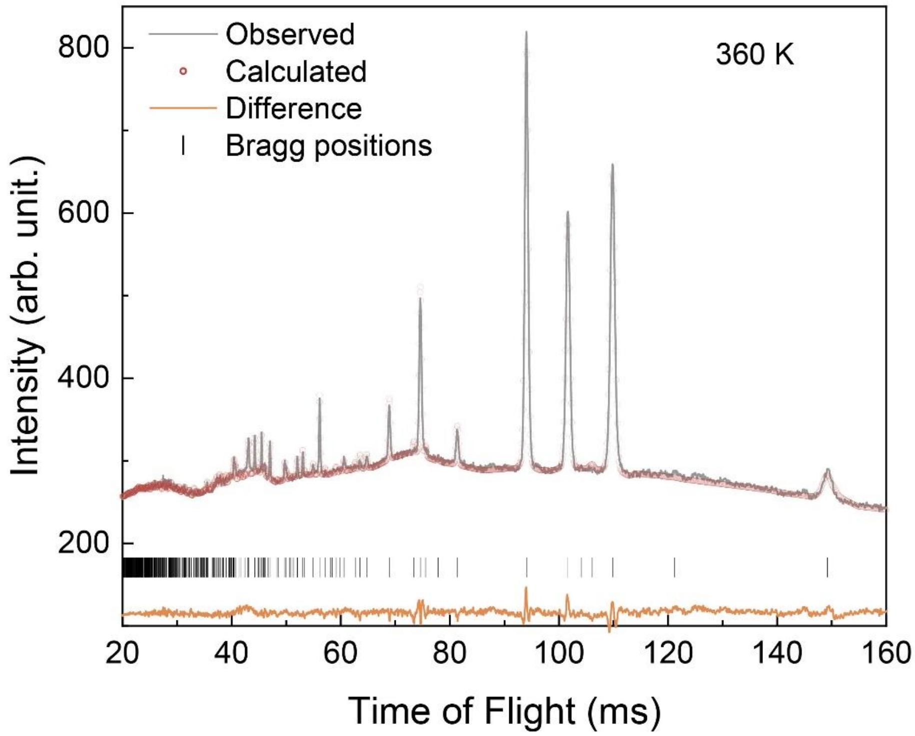

Supplementary Fig. 4. Rietveld refinement for the time-of-flight neutron diffraction profiles measured for $[Hdabco]ClO_4$ at 360 K.

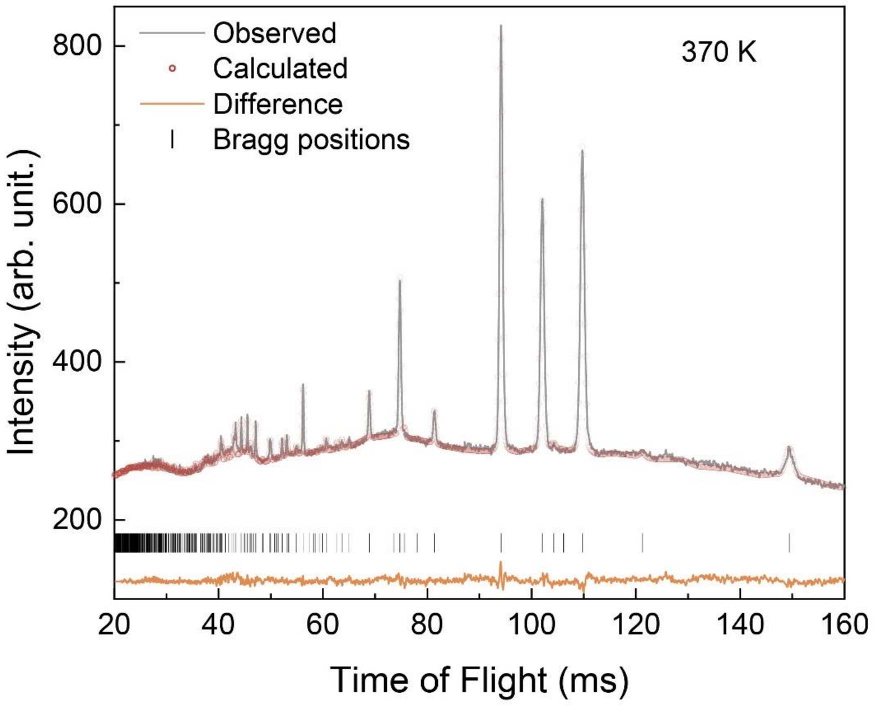

Supplementary Fig. 5. Rietveld refinement for the time-of-flight neutron diffraction profiles measured for $[Hdabco]ClO_4$ at 370 K.

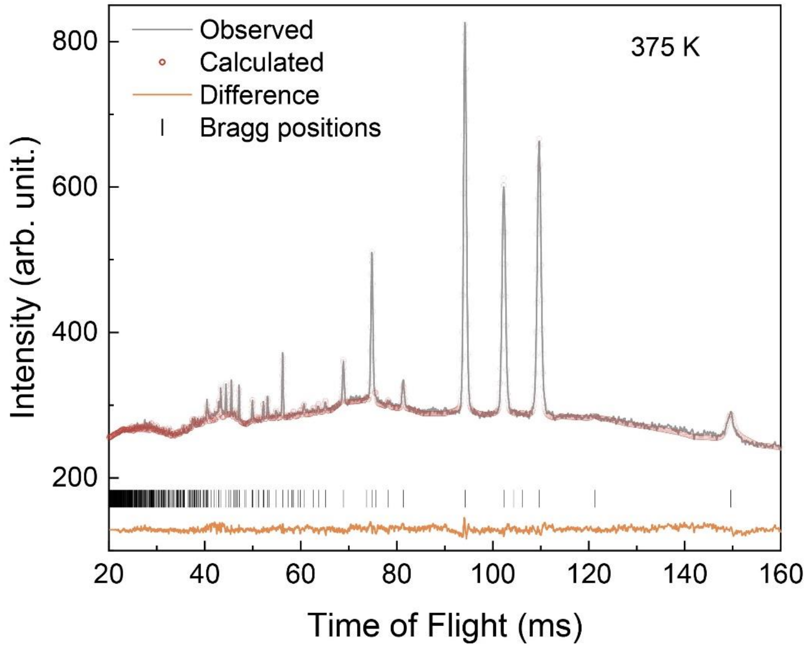

Supplementary Fig. 6. Rietveld refinement for the time-of-flight neutron diffraction profiles measured for $[Hdabco]ClO_4$ at 375 K.

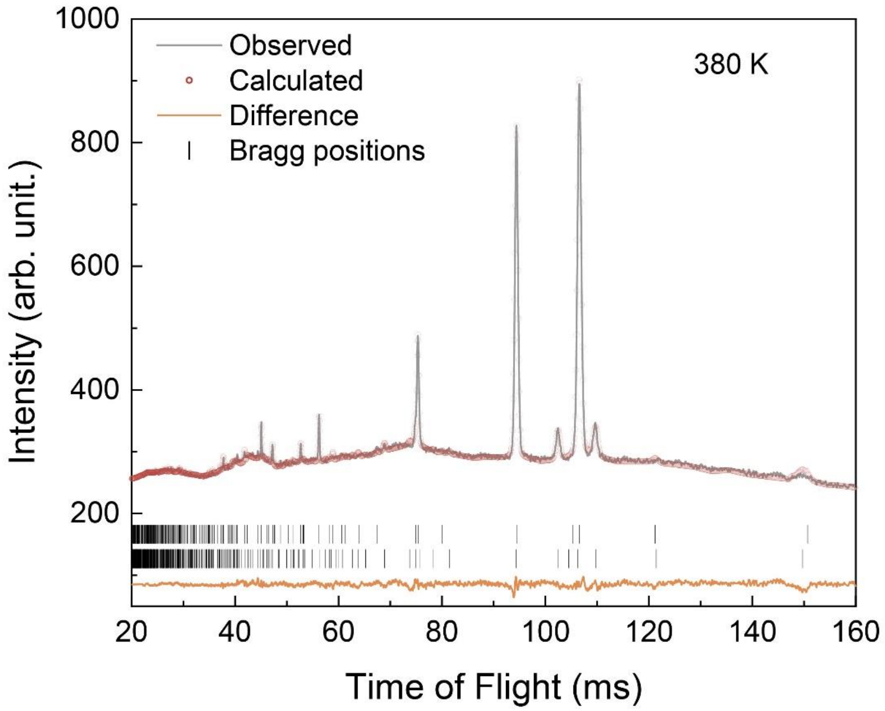

Supplementary Fig. 7. Rietveld refinement for the time-of-flight neutron diffraction profiles measured for [Hdabco]ClO_4 at 380 K.

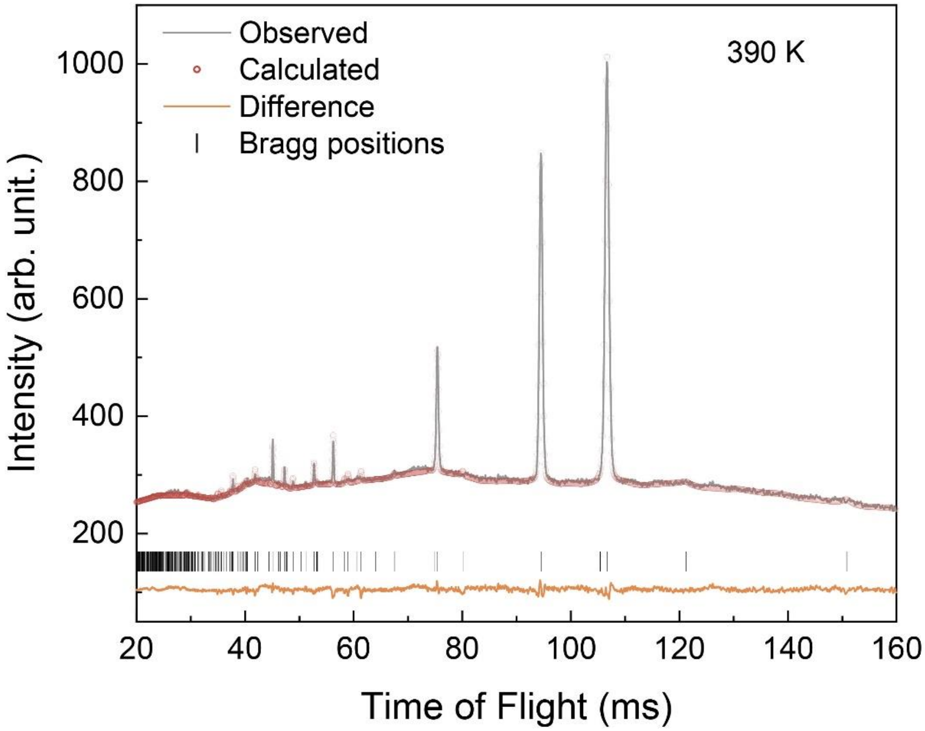

Supplementary Fig. 8. Rietveld refinement for the time-of-flight neutron diffraction profiles measured for [Hdabco]ClO_4 at 390 K.

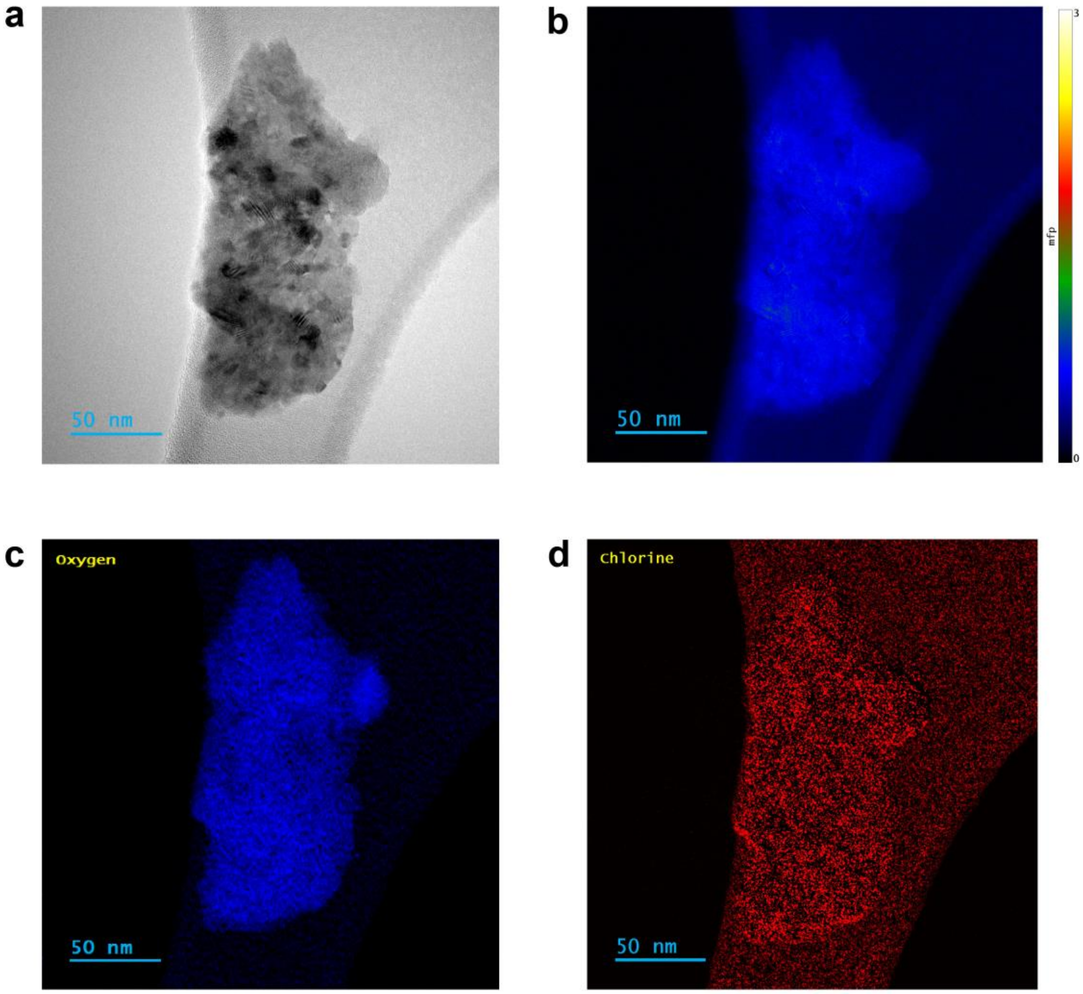

Supplementary Fig. 9. Multi-element maps for [Hdabco]ClO_4. **a**, Bright Field TEM image, **b**, thickness map, **c**, oxygen map, and **d**, chlorine map.

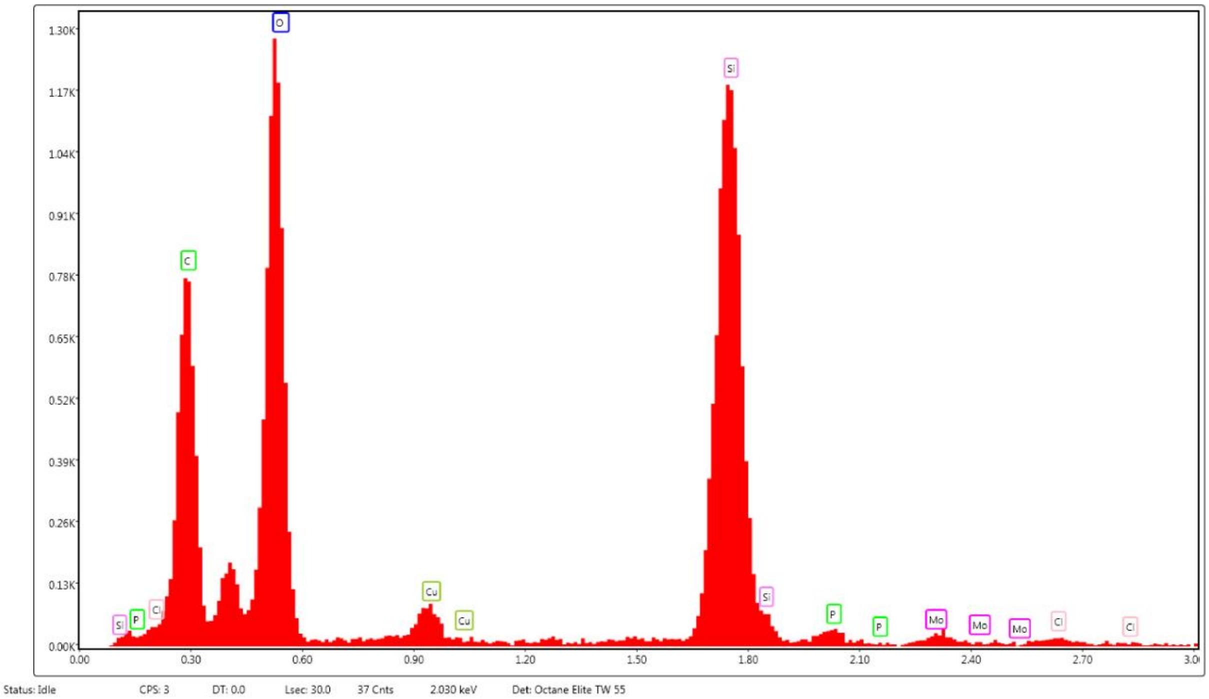

Supplementary Fig. 10. The X-ray energy dispersive spectrum for $[Hdabco]ClO_4$.

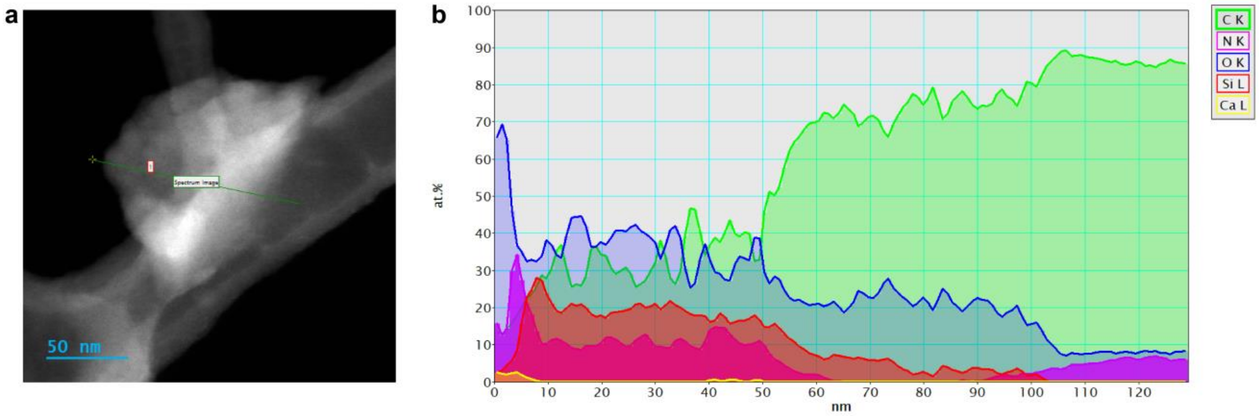

Supplementary Fig. 11. Chemical analyses of $[Hdabco]ClO_4$: **a**, high-angle annular dark-field (HAADF) image showing a line across the particle from the left to the right. **b**, count profile of each key element.

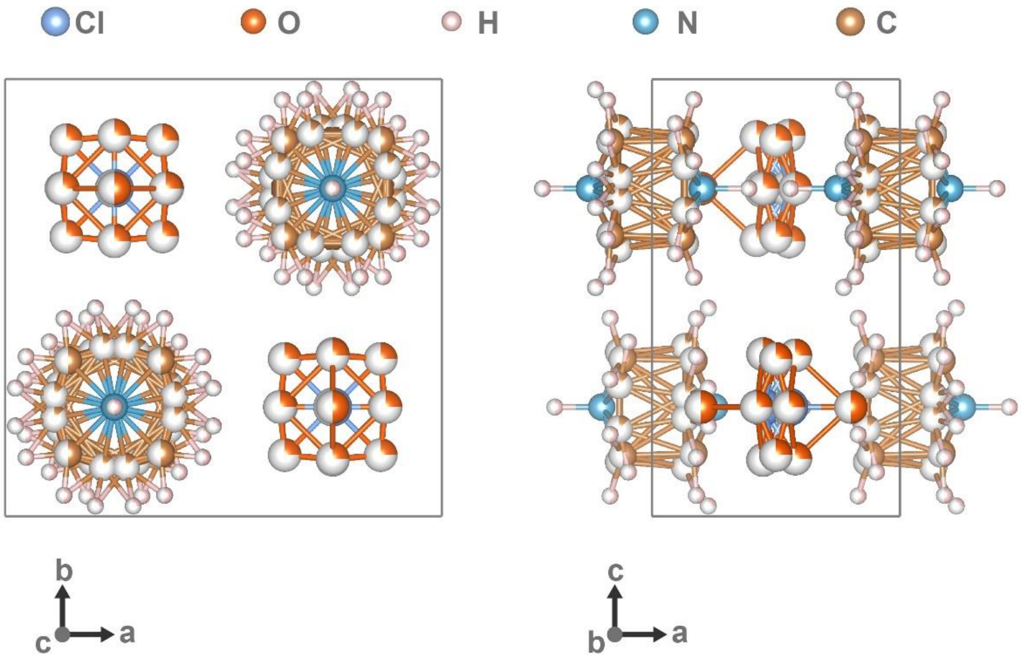

Supplementary Fig. 12. Crystal structure for the paraelectric phase of energetic [Hdabco]ClO_4.

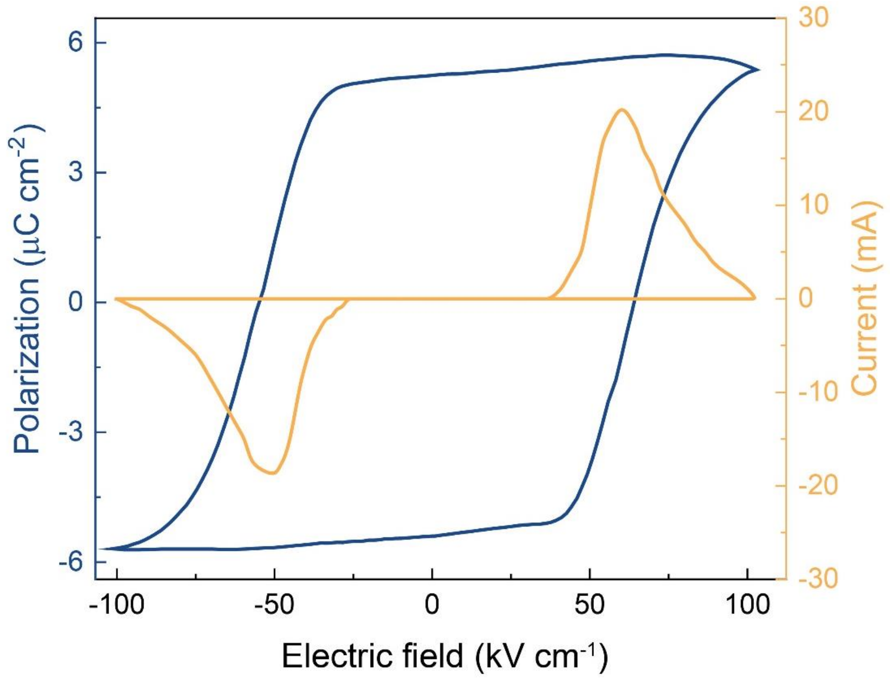

Supplementary Fig. 13. P-E and I-E loops of EIP at room temperature (100 Hz). The maximized current at the coercive field is known as direct evidence of ferroelectricity as the current peak is generated by the dipole reversal rather than electrical conductivity.

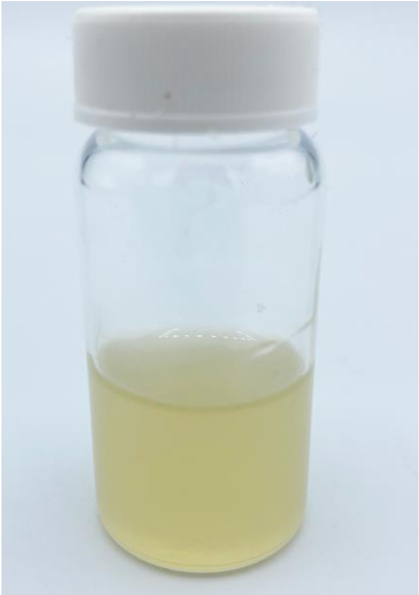

Supplementary Fig. 14. Optical image for the precursor including cellulose and dissolved [Hdabco]ClO_4.

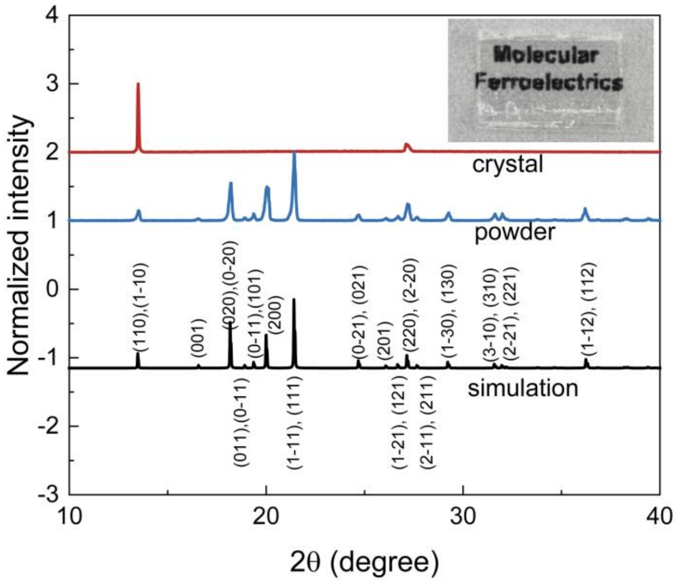

Supplementary Fig. 15. X-ray diffractions for [Hdabco]ClO_4 powder and self-assembled single crystal.

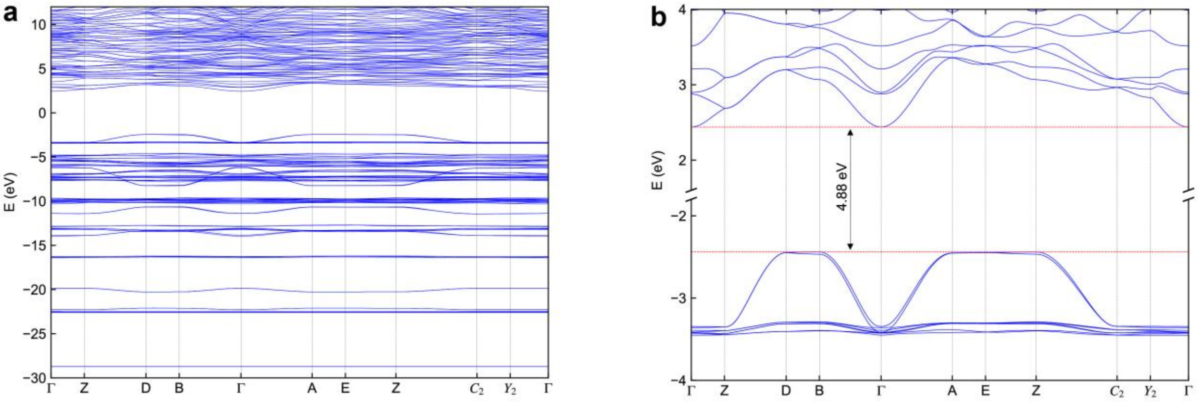

Supplementary Fig. 16. Calculated electronic band structure of [Hdabco]ClO_4. **a,** electronic band structure. **b,** zoomed-in plot for band-gap region.

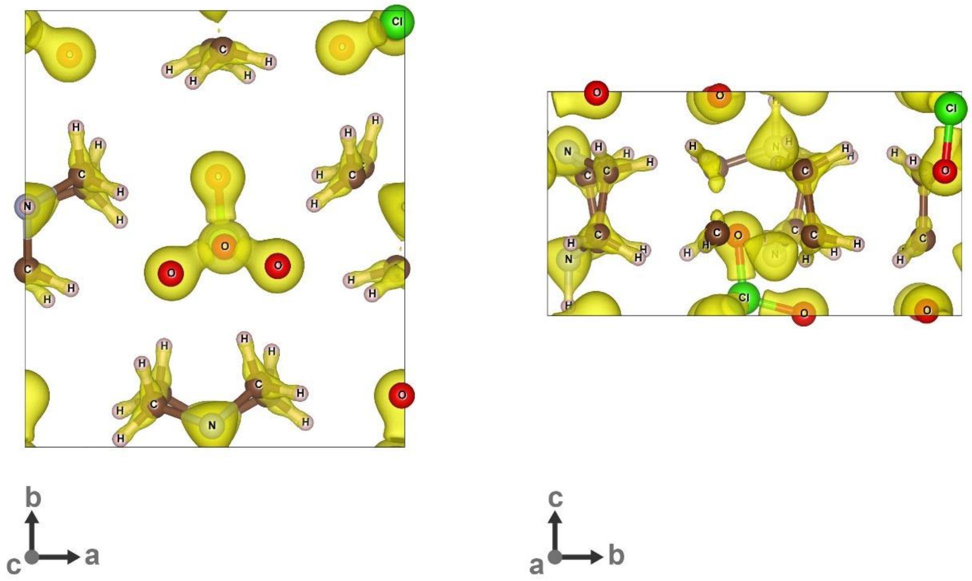

Supplementary Fig. 17. Calculated charge density of [Hdabco]ClO_4. **a,** Charge density along the c axis. **b,** Charge density along *a* axis**.**

The electronic structures are obtained based on DFT calculation. [Hdabco]ClO_4 shows an indirect band-gap of 4.88 eV. From the charge density of the structure, a higher density is observed around N and O atoms, showing the large electronegativity of those atoms. Moreover, the charge density is low around the H atoms of the N-H bonds and is high around the N atoms that are not attached to any H atoms. These could suggest the formation of hydrogen bonds (N−H···N) between the adjacent dabcoH+ cations, which have been observed in optical microscope photographs[8].

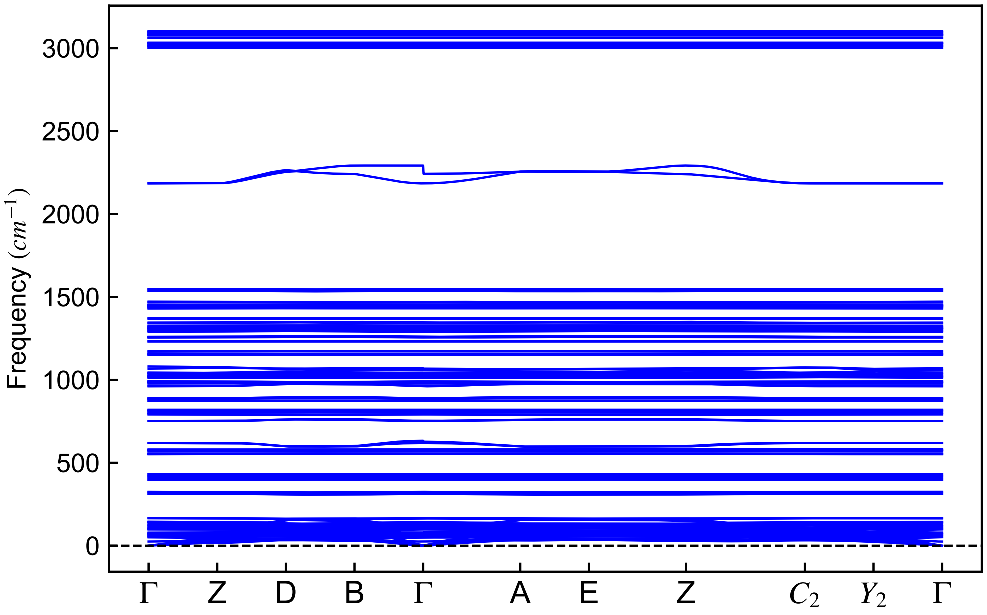

Supplementary Fig. 18. Calculated phonon dispersion for [Hdabco]ClO_4.

The experimentally reported structure of [Hdabco]ClO_4 crystal at 295K[8] is an orthorhombic unit cell which is used as our initial structure. The unit cell remains orthorhombic after geometry optimization. After an initial optimization, two phonon modes were found to have imaginary phonon frequencies of -100 cm^{-1}, which indicated the corresponding configuration is a saddle point of the energy surface. The eigenvectors of those phonon modes at the Γ point correspond to the motion of C and N atoms which result in the twist of the $dabcoH^+$ cation. By incrementally moving the atoms in the directions proportional to the directions in the eigenvectors, a double-well can be found. We then performed another geometry optimization using the configuration at the minimum of the double-well as the new starting point, and the final optimized structure was found to be monoclinic. The phonon dispersion of the monoclinic crystal only has negligible negative eigenvalues at the Γ point. It should also be noted that there is a discontinuity along A-Γ-B, which is also observed in other literature[9,10] and is likely due to the LO-TO splitting caused by the nonanalytical term correction[11]. The long-range nature of Coulomb interaction, together with the high polarizability of [Hdablo]ClO_4, make the high frequencies modes sensitive to the directions approaching the Γ point, resulting in different phonon frequencies near Γ along B-Γ and Γ-A.

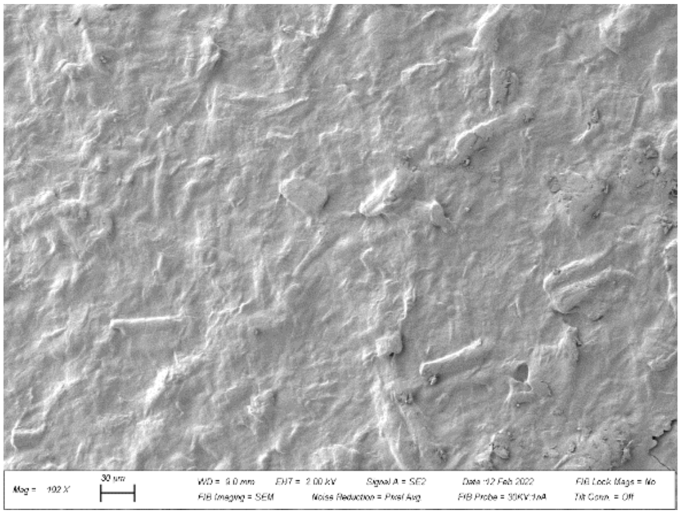

Supplementary Fig. 19. SEM images for dried cellulose from 333 K oven. The dried cellulose shows a dense structure.

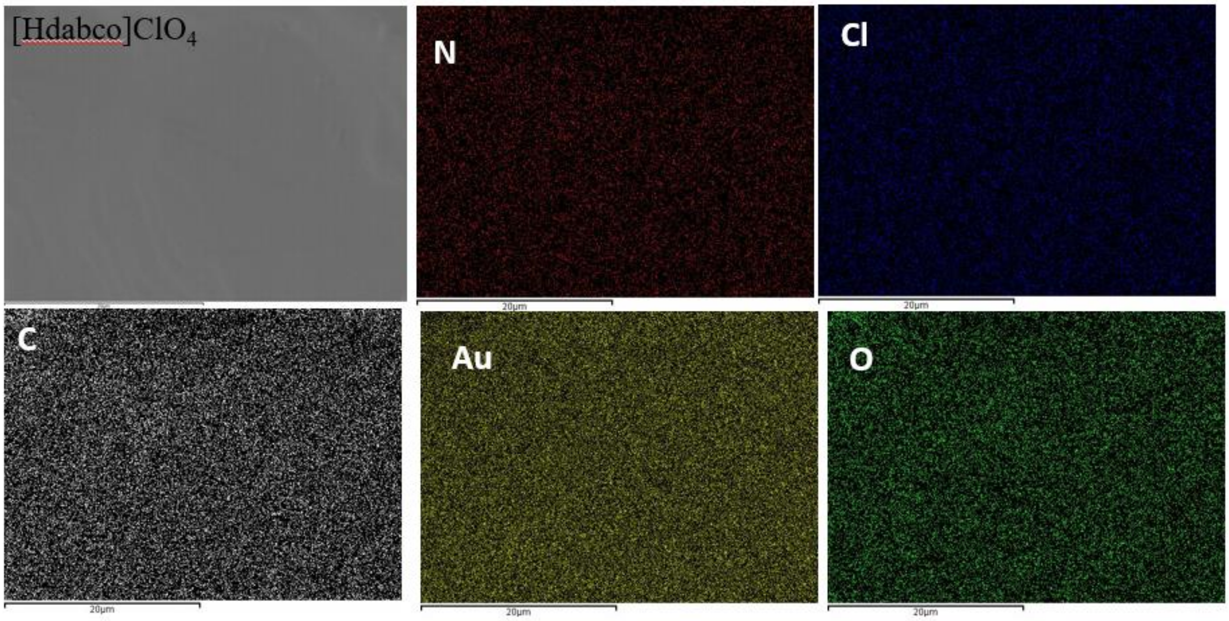

Supplementary Fig. 20. SEM images for surface of energetic [Hdabco]ClO_4 crystal. The pristine [Hdabco]ClO_4 crystal has a dense structure and smooth surface.

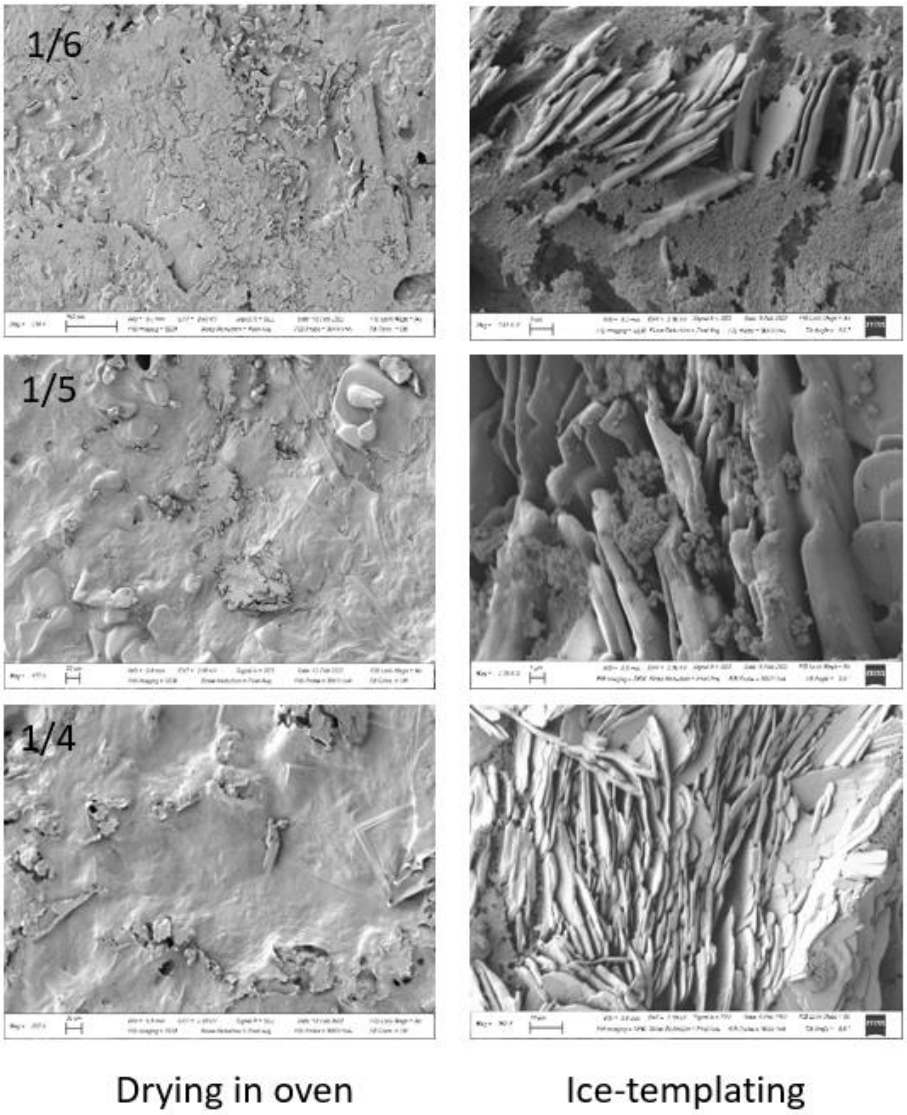

Supplementary Fig. 21. SEM images for 3D printed energetic [Hdabco]ClO_4 with different weight ratio (cellulose/ [Hdabco]ClO_4). Conventional drying method (left side) in a 333 K oven gives a dense structure. The ice-templating method (right side) results in an aligned architecture with a porous structures.

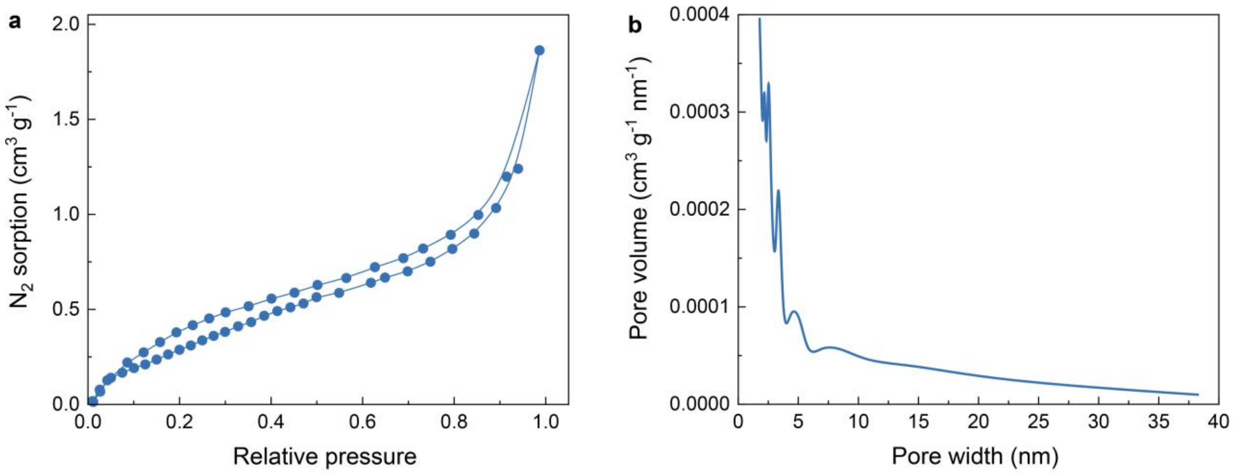

Supplementary Fig. 22. N_2 adsorption-desorption isotherms and the pore size distribution. **a**, Adsorption/desorption isothermal curve for 3D printed [Hdabco]ClO_4 (weight ratio=1/5) by Brunauer−Emmett−Teller (BET) theory. **b**, Pore size distribution for for 3D printed [Hdabco]ClO_4 (weight ratio=1/5) derived from Barrett–Joyner–Halenda.

High-pressure differential scanning colorimetry (HP-DSC) and pyrolysis-gas chromatography-mass spectrometry (GC/MS) study.

HP-DSC

Results for high-pressure DSC of [Hdabco]ClO_4 under N_2 show that enthalpy of decomposition increases with pressure but reach a maximum at approximately 200 psi (Supplementary Fig. 23a). The data clearly demonstrate that suppression of dabco volatilization by application of pressure results in a higher enthalpy of decomposition, presumably due to oxidation of dabco by O_2 from ClO_4^- decomposition.

DSC traces for cellulose, [Hdabco]ClO_4 and 3D printed [Hdabco]ClO_4 under 200 psi N_2 (Supplementary Fig. 23b and Fig. 4c (main text)) show that even when dabco volatilization is suppressed in both [Hdabco]ClO_4 and 3D printed [Hdabco]ClO_4 samples, the enthalpy of the 3D printed [Hdabco]ClO_4 still exceeds the calculated expected value.

A photo of the Al pan from HP-DSC analysis of [Hdabco]ClO_4 under 200 psi N_2 is given in Supplementary Fig. 24 and shows no evidence of oxidation of the pan itself.

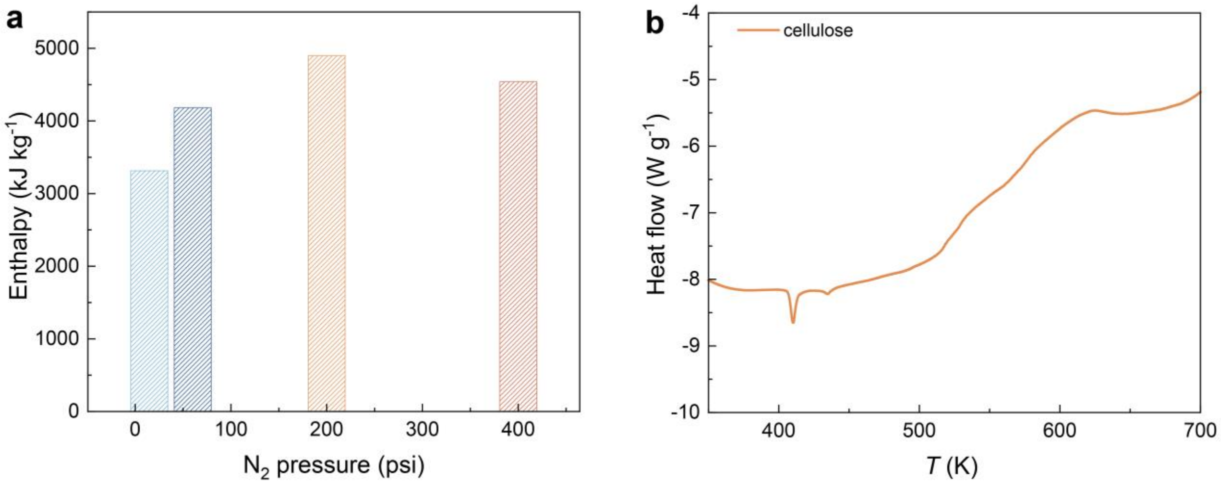

Supplementary Fig. 23. High-pressure differential scanning colorimetry study. **a,** N_2 pressure dependent enthalpy of decomposition for [Hdabco]ClO_4. **b**, HP-DSC traces for cellulose under 200 psi N_2. The calculated enthalpy of decomposition for cellulose under 200 psi N_2 is 2583 kJ kg^{-1}.

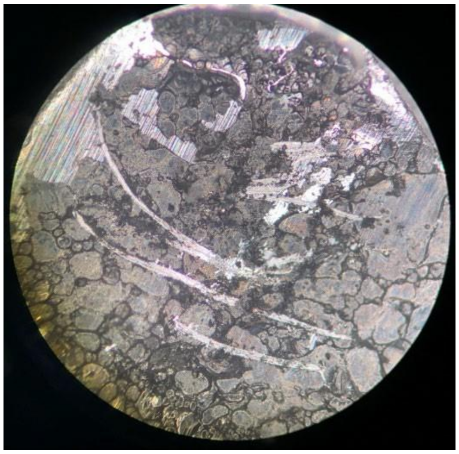

Supplementary Fig. 24. Photos of black residue in crimped Al pan after HP-DSC analysis of [Hdabco]ClO4 under 200 psi N_2. No evidence of oxidation or corrosion of the pan was observed. Scratches were deliberately made to determine if the residue was from reaction of the pan, or from the sample in the pan. The latter was the origin of the residue.

D/P-GC/MS

Results of D/P-GC/MS analysis of neat [Hdabco]ClO_4 and its cellulose composite are given in Supplementary Figs. 25-27 and Fig. 4d (main text); photos of the blackened residue remaining after analysis are given in Supplementary Fig. 28. Mass spectra for all figures are given in Supplementary Figs. 29-47.

While the thermal decomposition of [Hdabco]ClO_4 and 3D printed [Hdabco]ClO_4 is, for the most part, complete by 350 °C only dabco and its pyrazine pyrolysis products are observed at and below that temperature. Little evidence of dabco oxidation by the perchlorate is observed. Given that the oxygen balance for dabco is -185%, this is not very surprising. However, the lack of oxidation products also suggests that when in an unconfined condition under ambient pressure, the fuel component of the [Hdabco]ClO_4 readily diffuses away from the ClO_4^- oxidant before reaction can occur. In such a situation, the full energetic potential of a material cannot be realized.

Even though oxidation products are not observed in either [Hdabco]ClO_4 or 3D printed [Hdabco]ClO_4, it is noted from the 350 °C pyrolysis results that the 3D printed [Hdabco]ClO_4 behaves differently than the [Hdabco]ClO_4, i.e., the 3D printed [Hdabco]ClO_4 has a higher ratio of intact dabco relative to pyrazine decomposition products suggesting that cellulose somehow influences the distribution of dabco-related products. Assuming that dabco decomposition proceeds through a radical mechanism, it is likely that dabco

fragment radicals react with the cellulose matrix, thereby contributing to the black residual material observed at the conclusion of the analysis (Supplementary Fig. 28).

GC/MS result are also telling in that they show very little with respect to chlorine-containing volatiles. For any perchlorate, one would expect to see significant levels of HCl or Cl_2 among the products. While a relatively small amount of HCl is observed when a SIC for m/z = 36 is extracted from the 400 °C pyrolysis results for the 3D printed [Hdabco]ClO_4, the level is far below that expected. It is proposed that Cl radicals formed on decomposition of ClO_4^- reacted with available fuel (i.e., the cellulose matrix and any dabco that did not evaporate) and contributed to the observed black residue.

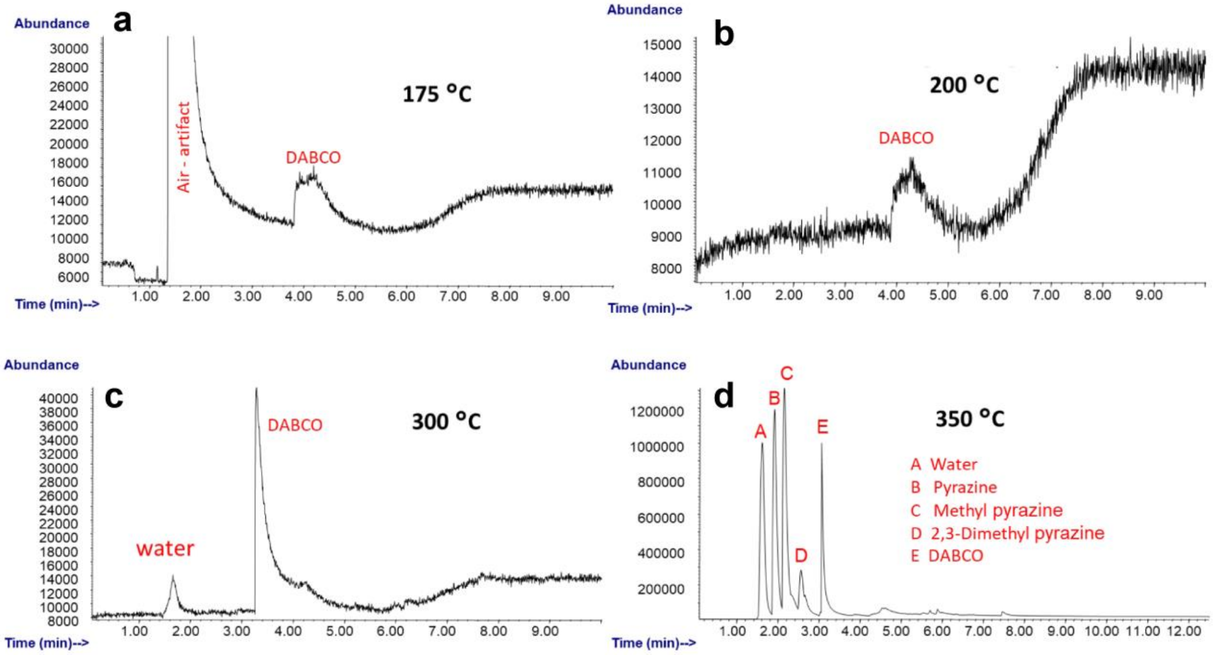

Supplementary Fig. 25. Total ion chromatograms for sequential desorption/pyrolysis of [Hdabco]ClO_4 at **a,** 175 °C, **b,** 200 °C, **c,** 250 °C and **d,** 350 °C.

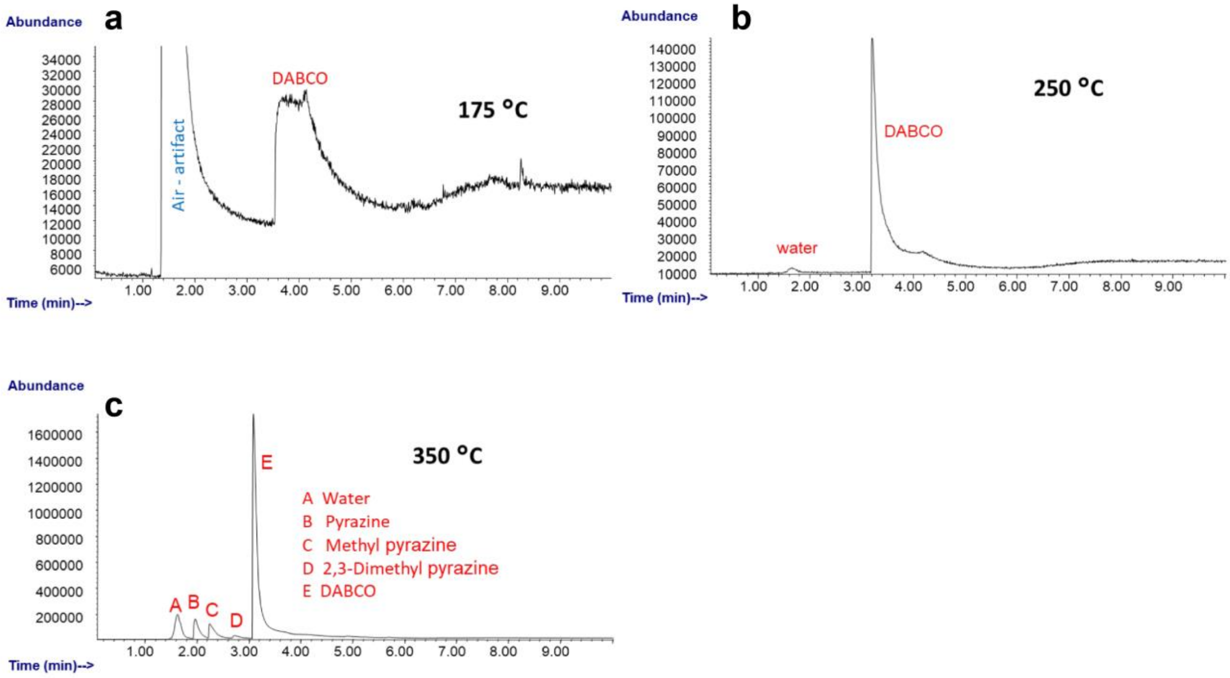

Supplementary Fig. 26. Total ion chromatograms for sequential desorption/pyrolysis of 3D printed [Hdabco]ClO_4 at **a,**175 °C, **b,** 250 °C and **c,** 350 °C.

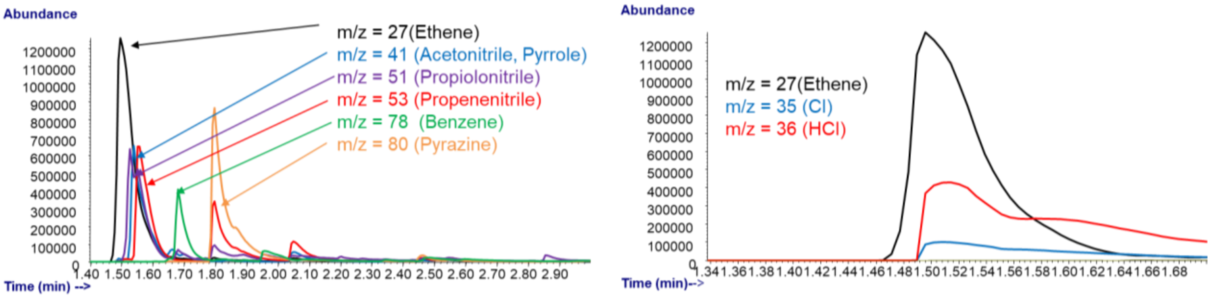

Supplementary Fig. 27. Selected ion chromatograms for pyrolysis of 3D printed [Hdabco]ClO_4 at 400°C. The red arrow in the total ion chromatogram indicates the maximum Y-axis value of the two selected ion chromatograms. M/z values are major mass spectral peaks for the species indicated in parentheses. M/z = 70 (Cl_2), was not observed to an appreciable extent and is not shown.

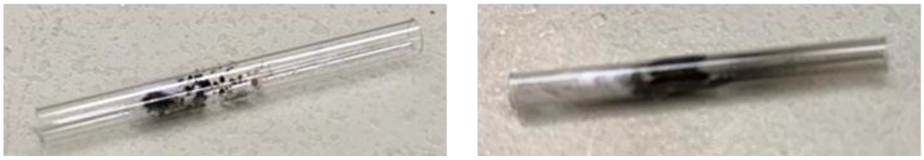

Supplementary Fig. 28. Photos of black residue in quartz tubes after 350°C pyrolysis of [Hdabco]ClO_4 (left) and 400°C pyrolysis of 3D [Hdabco]ClO_4 (Right).

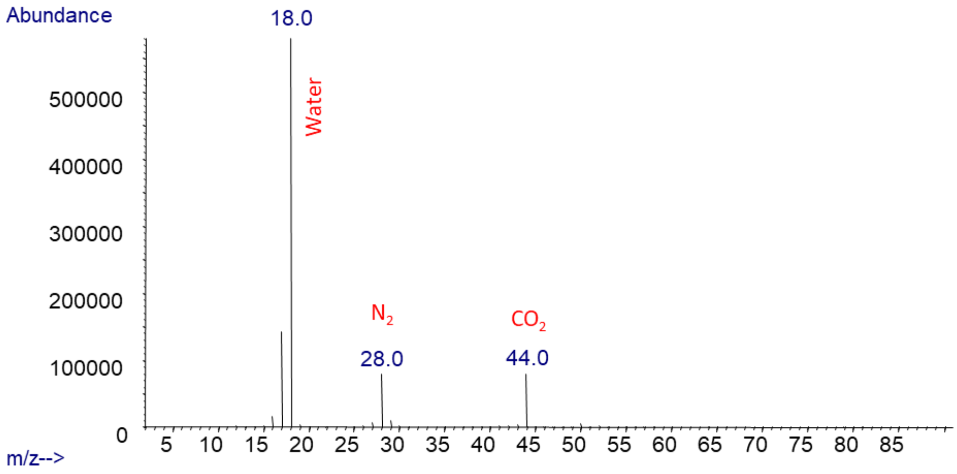

Supplementary Fig. 29. Mass spectrum of 1.631 min peak in Supplementary Fig. 25d.

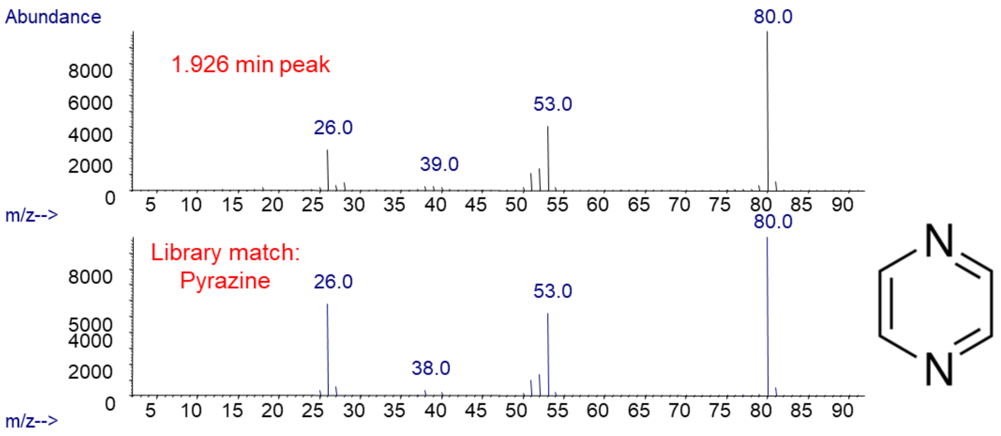

Supplementary Fig. 30. Mass spectrum of 1.926 min peak in Supplementary Fig. 25d and library search match (pyrazine).

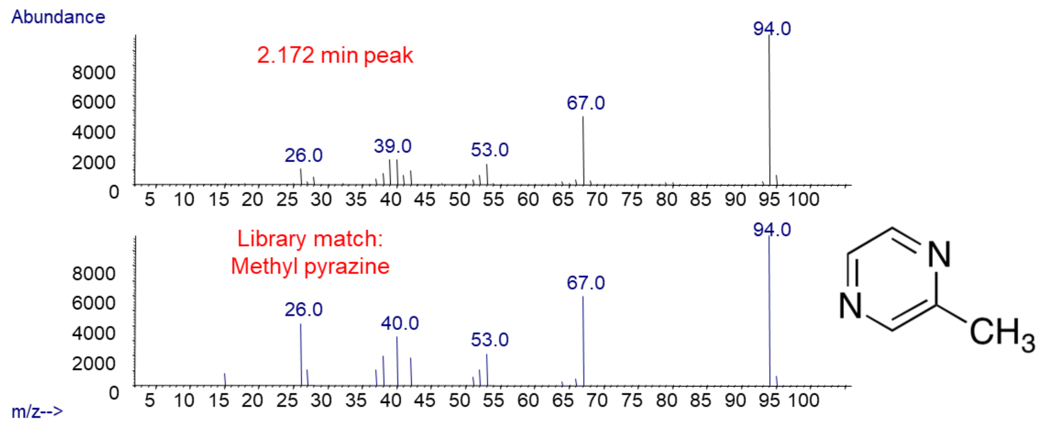

Supplementary Fig. 31. Mass spectrum of 2.172 min peak in Supplementary Fig. 25d and library search match (methyl pyrazine).

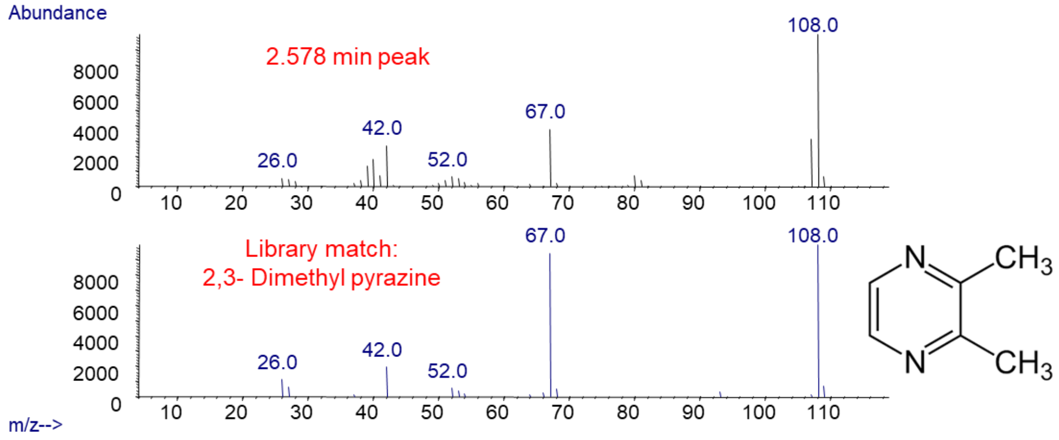

Supplementary Fig. 32. Mass spectrum of 2.578 min peak in Supplementary Fig. 25d and library search match (2,3-dimethyl pyrazine).

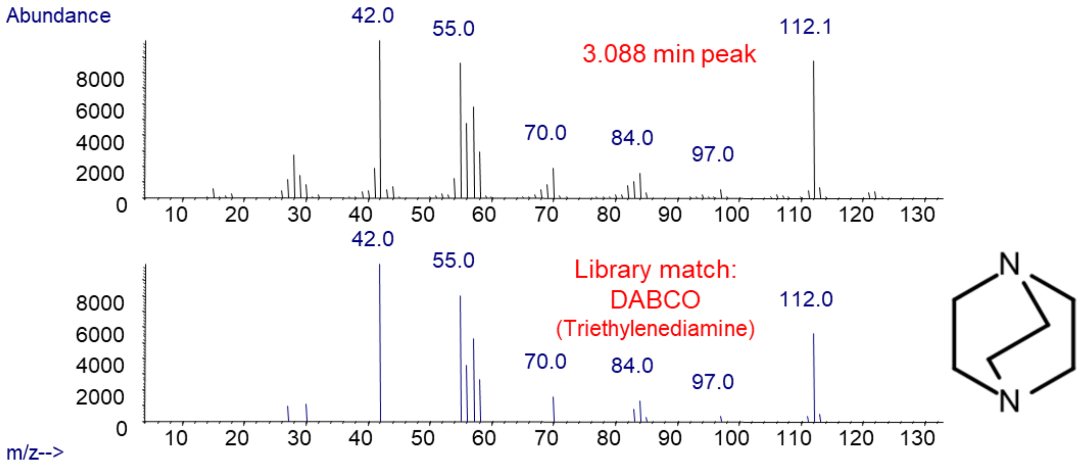

Supplementary Fig. 33. Mass spectrum of 3.088 min peak in Supplementary Fig. 25d and library search match (DABCO).

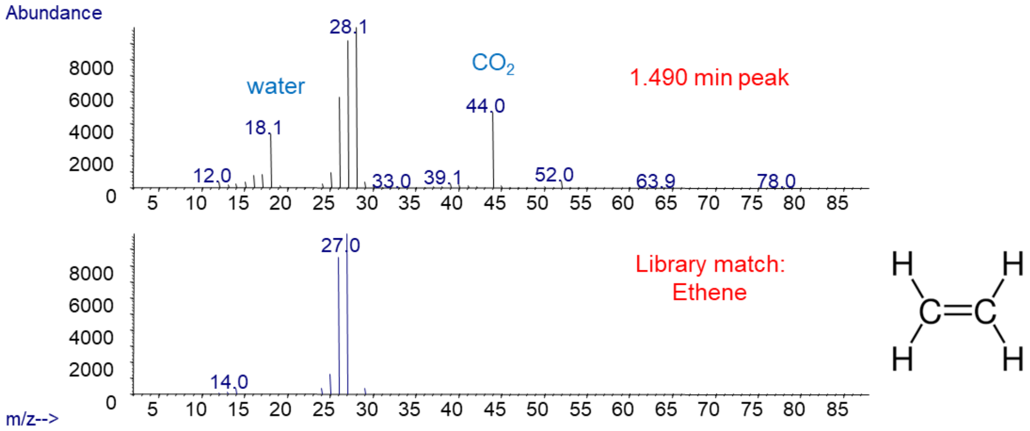

Supplementary Fig. 34. Mass spectrum of 1.490 min peak in Supplementary Fig. 26c and library search match (ethylene).

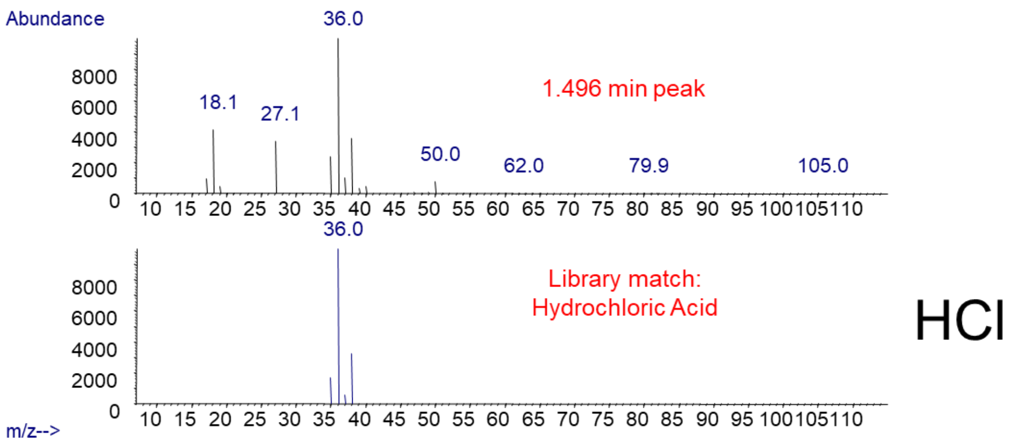

Supplementary Fig. 35. Mass spectrum of 1.496 min peak in Supplementary Fig. 26c and library search match (HCl).

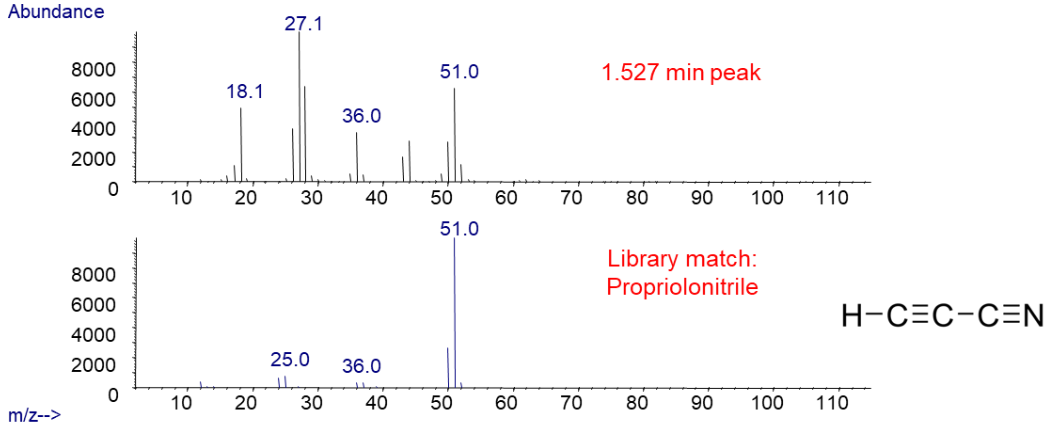

Supplementary Fig. 36. Mass spectrum of 1.527 min peak in Supplementary Fig. 26c and library search match (propiolonitrile).

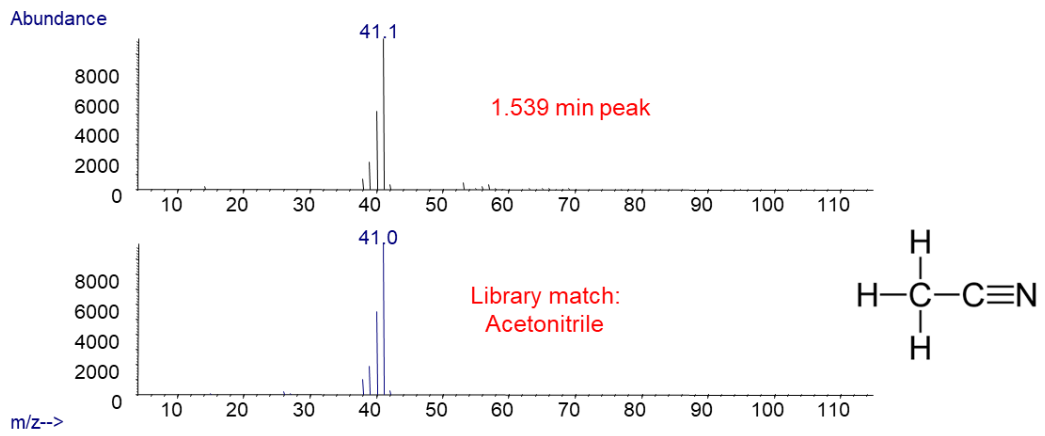

Supplementary Fig. 37. Mass spectrum of 1.539 min peak in Supplementary Fig. 26c and library search match (acetonitrile).

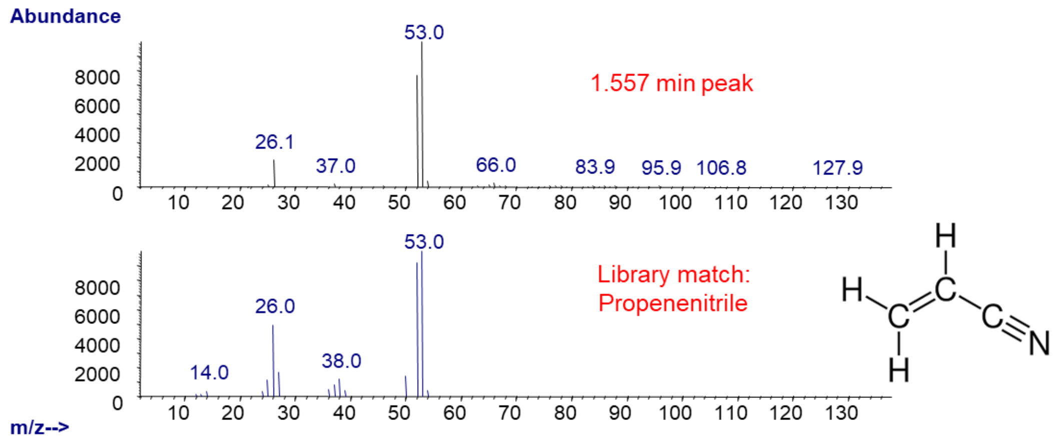

Supplementary Fig. 38. Mass spectrum of 1.557 min peak in Supplementary Fig. 26c and library search match (propenenitrile).

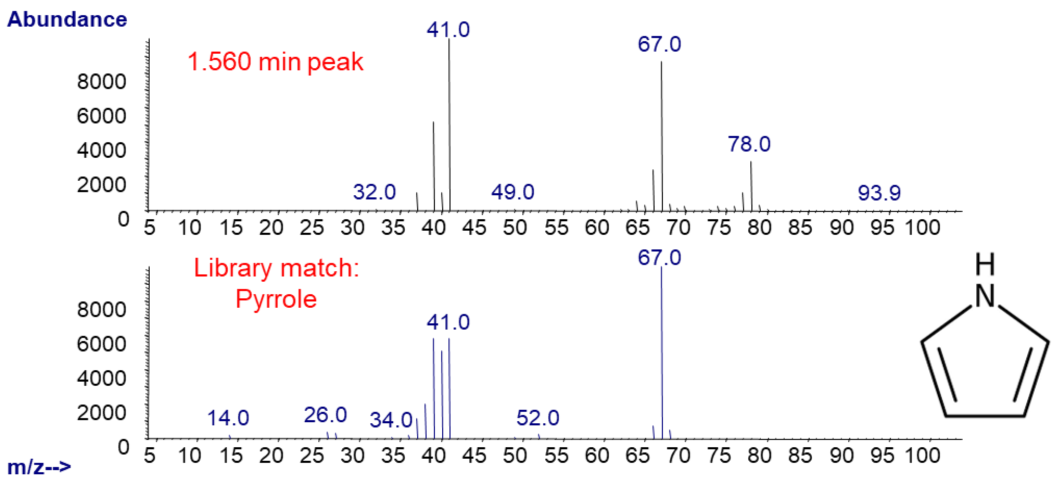

Supplementary Fig. 39. Mass spectrum of 1.560 min peak in Supplementary Fig. 26c and library search match (pyrrole).

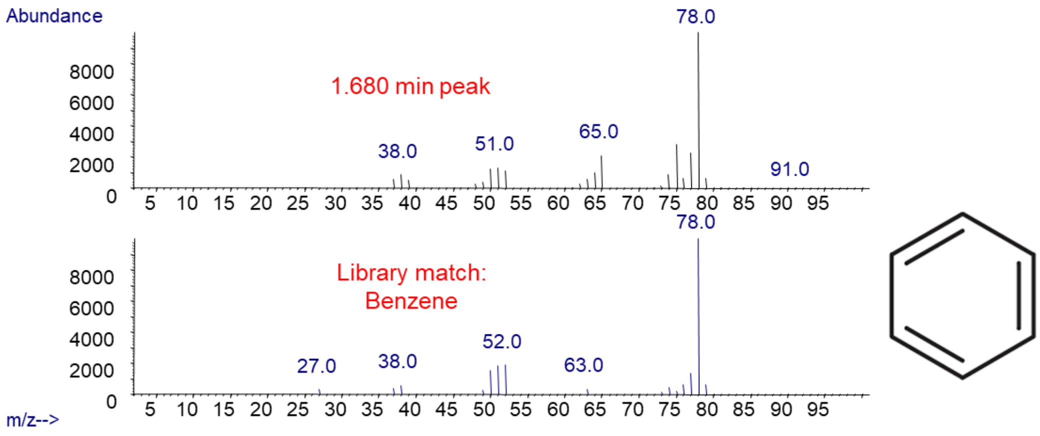

Supplementary Fig. 40. Mass spectrum of 1.680 min peak in Supplementary Fig. 26c and library search match (benzene).

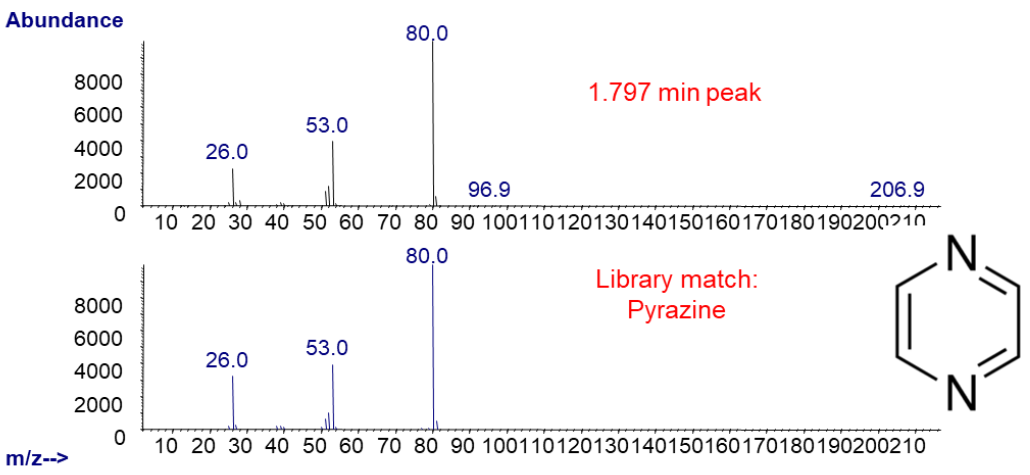

Supplementary Fig. 41. Mass spectrum of 1.797 min peak in Supplementary Fig. 26c and library search match (pyrazine).

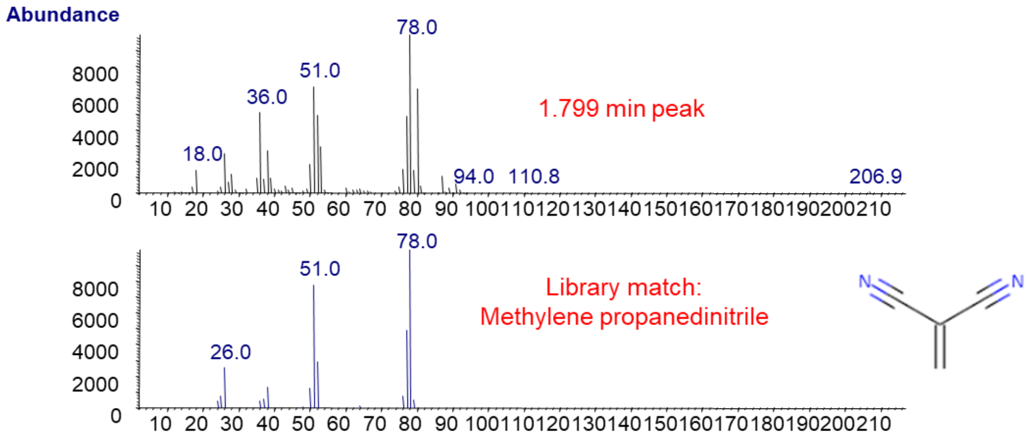

Supplementary Fig. 42. Mass spectrum of 1.799 min peak in Supplementary Fig. 26c and library search match (methylene propanedinitrile).

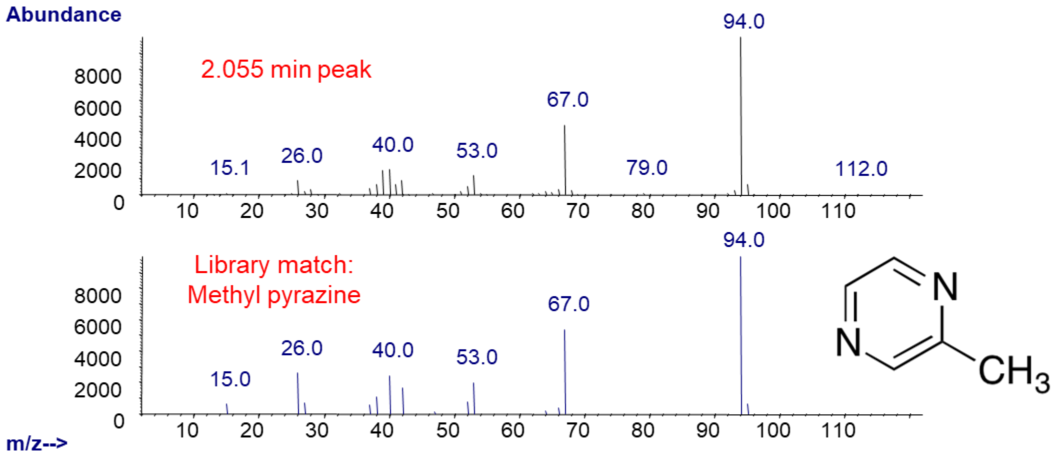

Supplementary Fig. 43. Mass spectrum of 2.055 min peak in Supplementary Fig. 26c and library search match (methyl pyrazine).

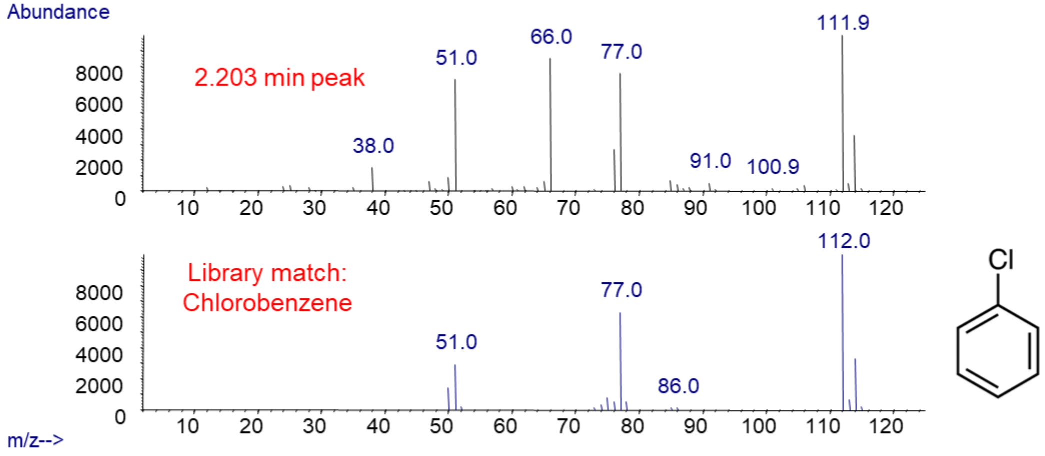

Supplementary Fig. 44. Mass spectrum of 2.203 min peak in Supplementary Fig. 26c and library search match (chlorobenzene).

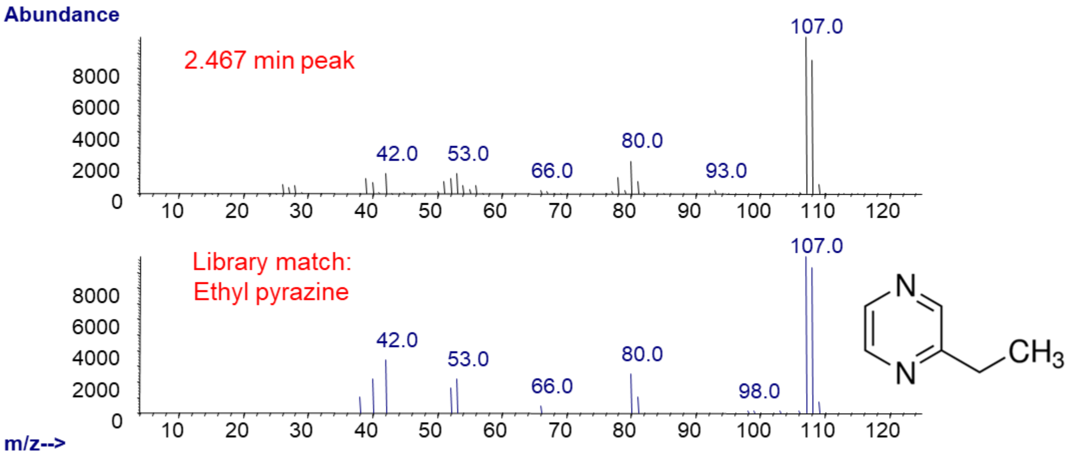

Supplementary Fig. 45. Mass spectrum of 2.467 min peak in Supplementary Fig. 2c and library search match (ethyl pyrazine).

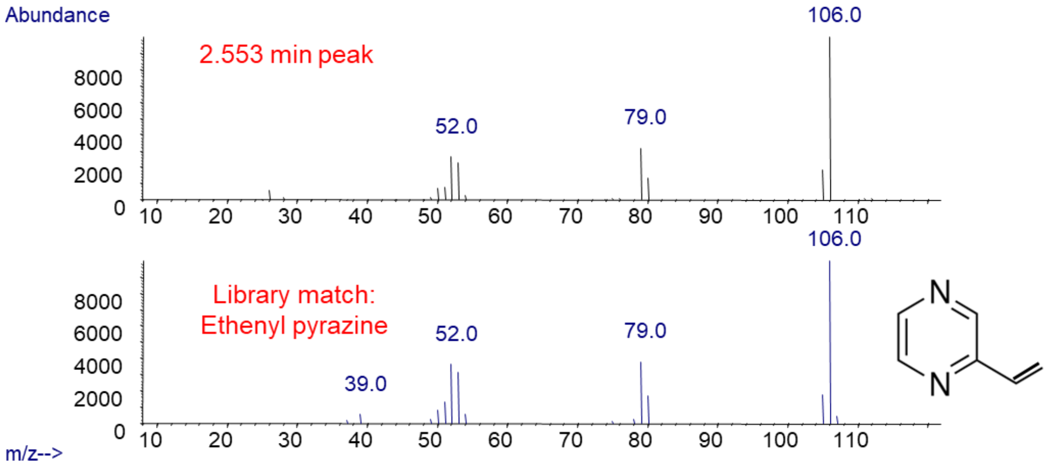

Supplementary Fig. 46. Mass spectrum of 2.553 min peak in Supplementary Fig. 26c and library search match (ethenyl pyrazine).

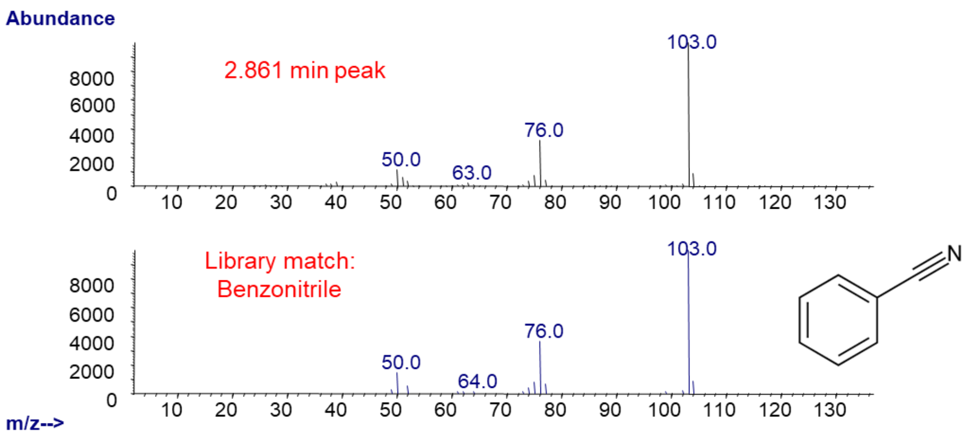

Supplementary Fig. 47. Mass spectrum of 2.861 min peak in Supplementary Fig. 26c and library search match (benzonitrile)

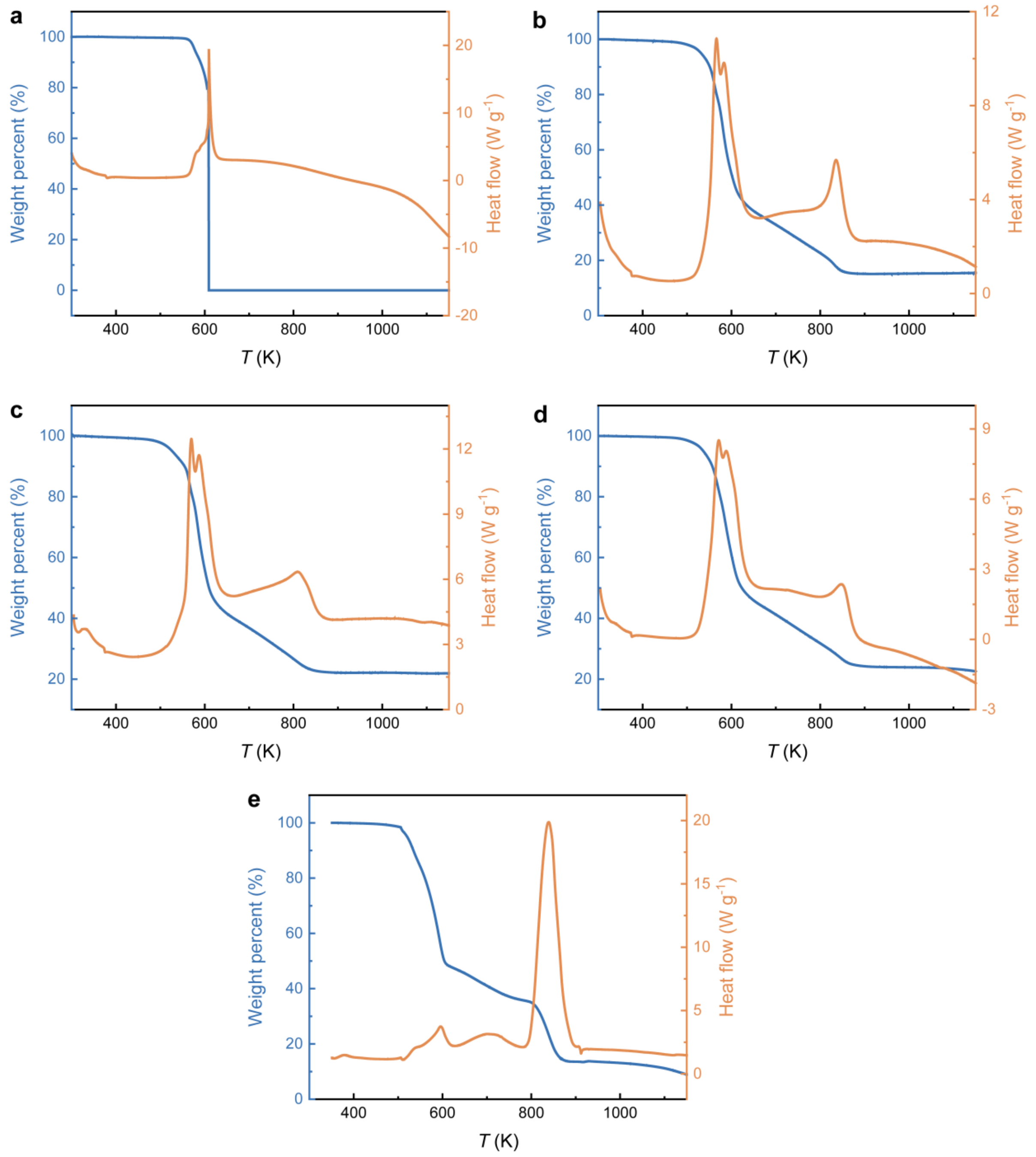

Supplementary Fig. 48. Thermogravimetric analysis and differential scanning calorimetry measurements for 3D printed energetic [Hdabco]ClO_4 with different weight ratio (cellulose/[Hdabco]ClO_4). **a**, weight ratio=0, **b**, weight ratio=1/6, **c**, weight ratio=1/5, **d**, weight ratio=1/3. The heat of decomposition is 1509, 6174, 6180, and 6162 kJ kg^{-1} for the weight ratio (cellulose/[Hdabco]ClO_4) of 0, 1/6, 1/5, and 1/3, respectively. **e**, Thermogravimetric analysis and differential scanning calorimetry measurements for cellulose.

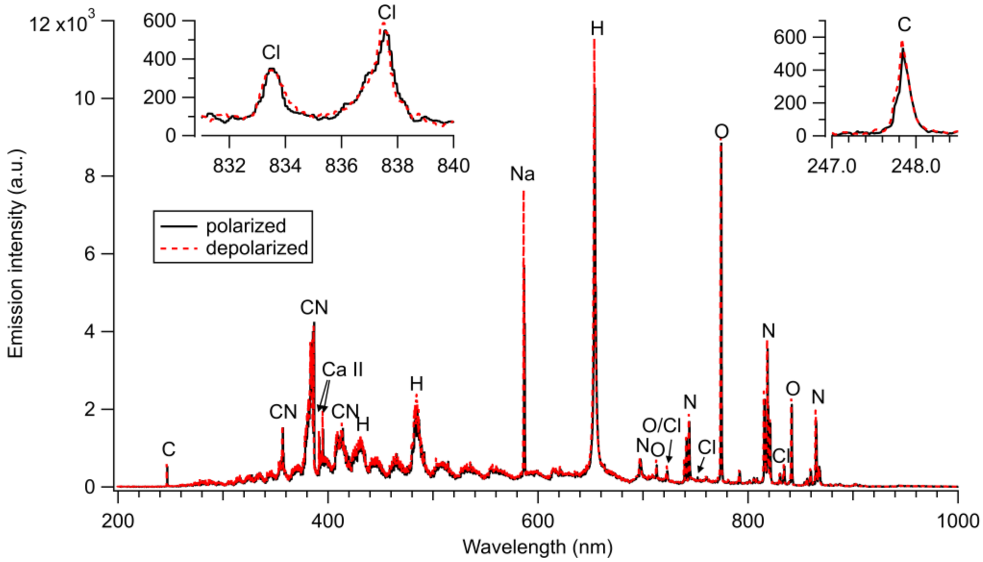

Supplementary Fig. 49. The plasma emission spectrum for poled and unpoled 3D printed [Hdabco]ClO_4 (weight ratio=1/5) measured during the LASEM experiments.

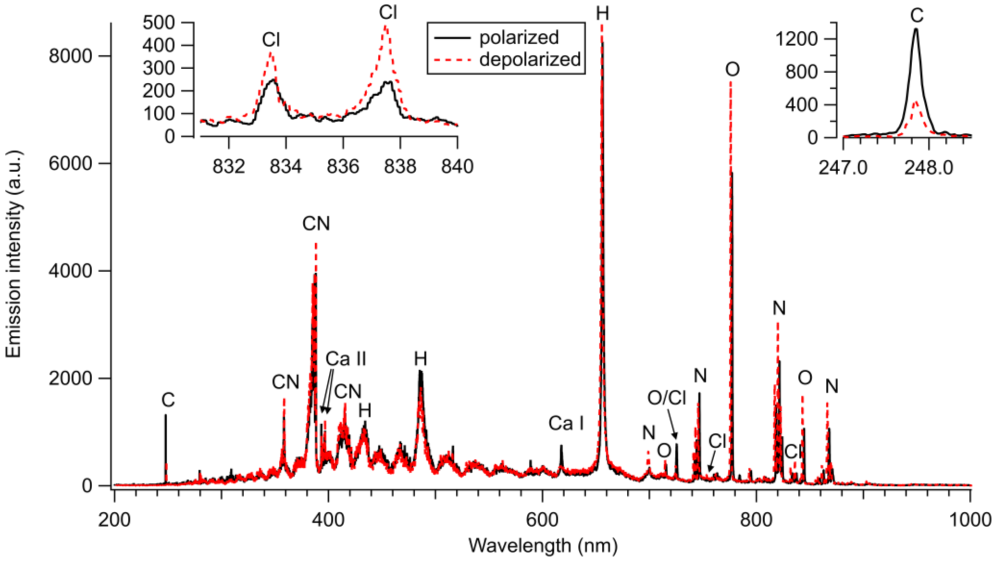

Supplementary Fig. 50. The plasma emission spectrum for poled and unpoled [Hdabco]ClO_4 measured during the LASEM experiments.

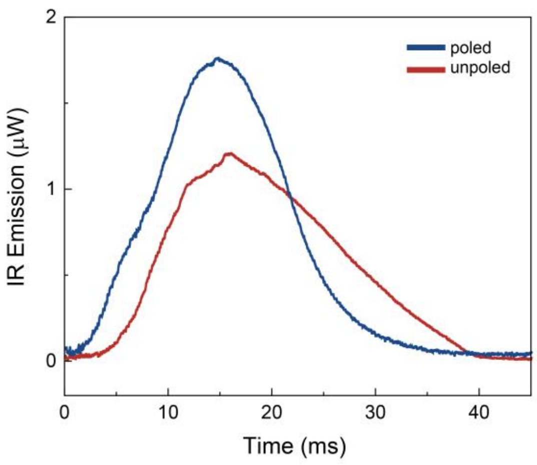

Supplementary Fig. 51. The time-resolved IR emission for poled and unpoled [Hdabco]ClO_4 measured during the LASEM experiments, indicating the extent of combustion with air on the millisecond timescale.

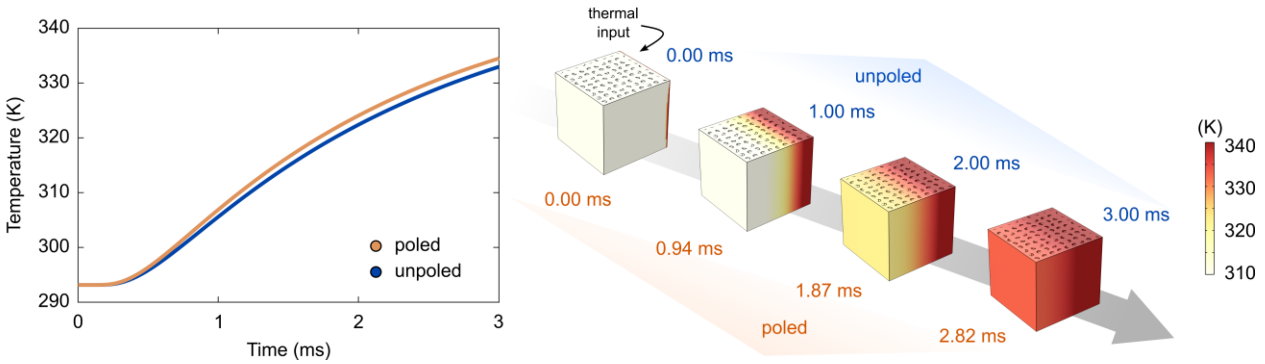

Supplementary Fig. 52. Thermal response simulation for [Hdabco]ClO_4 composite.

The computational finite element model is comprised of a 30 μm cubic [Hdabco]ClO_4 matrix housing 81 hollow pillars (i.e., pores) with an average stacking density of 3 pillars/10 μm. The pillars are formed using a spline with four points, one on the base, two in the middle, and one on the top surface all selected via a Gaussian distribution. Cellulose nanofibers are created using line elements centered at the two middle spline points and a length slightly larger than the diameter of the hollow pillars. The line edges are selected using a polar format with fixed length and randomly selected azimuth and elevation angles. All the elements are free tetrahedral. The thermal response simulation is carried out by using the "Heat Transfer in Solids" module of COMSOL Multiphysics. The measured thermal conductivity (Fig. 5f of main text) is used for the simulation. A thermal input is applied using a boundary heat source (100 W m^{-2} heat flux) and an initial temperature of 340 K at the input location indicated on the figure. A time domain simulation of the thermal response is then conducted and the surface temperature at the opposing boundary is measured over the first 3 milliseconds with a time step of $3e^{-6}$ seconds.

Supplementary Table S1. Comparison of five different featurization methods for prediction of detonation velocity with various combinations of machine learning models.

Hyperparameter optimization was used with 5-fold cross validation. The Mean Absolute Error (MAE) is averaged over 3 train-test sets with 2/1 splitting. The models are Gaussian Process Regression (GP), Kernel Ridge Regression (KRR), Support Vector Regression (SVR), Random Forest (RF), Lasso Regression (Lasso), k-Nearest Neighbors (kNN), Gradient Boosting (GBoost) and Ridge Regression (Ridge). Estate: E-State Fingerprint, CDS: Custom Descriptor Set, SoB: Sum Over Bonds.

Input Feature	Model	MAE (km s^{-1})
Estate + CDS + SoB	GP	0.83
	KRR	0.98
	SVR	1.12
	RF	1.26
	Lasso	0.99
	KNN	0.96
	GBoost	1.21
	Ridge	0.98
Estate	GP	0.83
	KRR	0.60
	SVR	0.64
	RF	1.25
	Lasso	0.64
	KNN	1.3
	GBoost	1.06
	Ridge	0.55
CDS	GP	0.75
	KRR	1.08
	SVR	0.97
	RF	1.34
	Lasso	0.90
	KNN	1.00
	GBoost	1.12
	Ridge	0.99
SoB	GP	0.85
	KRR	0.76
	SVR	0.98
	RF	0.89
	Lasso	0.70
	KNN	1.12
	GBoost	1.38
	Ridge	0.85
Joint Embedding with Estate + CDS + SoB	GP	0.92
	KRR	1.57
	SVR	1.16
	RF	0.70
	Lasso	1.76
	KNN	1.10
	GBoost	0.68
	Ridge	1.48

Supplementary Table S2. Predicted detonation velocity of water-soluble molecular ferroelectrics.

With Ridge regression and Estate fingerprint, we predict the detonation velocity of each water-soluble ferroelectric material in the dataset. As noted, in this phase of model predictions, the model is trained with 401 energetic materials.

**Molecular Ferroelectrics**	***T_c* (K)**	**SMILES**	**Predicted Detonation Velocity (km s^{-1})**
Triglycine sulfate (TGS)	322	S(=O)(=O)(O)O.NCC(=O)O.NCC(=O)O.NCC(=O)O	6.50509896
trimethylbromomethylammonium tribromomanganese(II) (TMBM-$MnBr_3$)	415	Br[Mn-](Br)Br.C[N+](CBr)(C)C	6.49595066
[Hdabco]ClO_4<br>Hdabco = monoprotonated 1,4-diazabicyclo[2.2.2]octane	377	C1CN2CC[NH+]1CC2.[O-]Cl(=O)(=O)=O	6.71119793
[Et_4N]ClO_4; Et_4N = tetraethylammonium	378	CC[N+](CC)(CC)CC.[O-]Cl(=O)(=O)=O	5.30865801
[qui]IO_4; qui = quinuclidinium	322	I(=O)(=O)(=O)[O-].[NH+]12CCC(CC1)CC2	6.44097404
[hqu]Cl<br>hqu = (R)-(−)-3-hydroxlyquinuclidinium	340	C1C[NH+]2CCC1C(C2)O.[Cl-]	6.54473404
[MeHdabco]RbI_3<br>MeHdabco = protonated N-methyl-1,4-diazoniabicyclo[2.2.2]octane	430	[I-].[I-].[I-].[Rb+].C[N+]12CC[NH+](CC1)CC2	6.13072686
[tmno]$_2$[KFe$(CN)_6$]<br>tmno = protonated trimethylamine N-oxide	402	[Fe-3](C#N)(C#N)(C#N)(C#N)(C#N)C#N.[K+].O[N+](C)(C)C	6.42713375
[tmcm]$MnCl_3$<br>tmcm = trimethylchloromethylammonium	406	[Cl-].[Mn+3].C[N+](CCl)(C)C.[Cl-].[Cl-].[Cl-]	6.49595066
Diisopropylammonium bromide (DIPAB)	426	CC(C)[NH2+]C(C)C.[Br-]	6.04530262
1-Azabicyclo[2.2.1]heptanium perrhenate ([AH][ReO_4])	322	[Re](=O)(=O)(=O)[O-].[NH+]12CCC(CC1)C2	6.44450487
tetramethylammonium tetrachloroferrate(III) [$(CH_3)_4$ $NFeCl_4$]	344	C[N+](C)(C)C.[Cl-].Cl[Fe](Cl)Cl	6.30495223
Croconic acid	420	C1(=C(C(=O)C(=O)C1=O)O)O	6.49595066

Supplementary Table S3. Hyperparameters chosen for different machine learning models.

**Model**	**'Hyperparameter Name': Range of Hyperparameter**
GP	'length_scale': np.linspace(0.1, 5., 2),<br>'nu': np.linspace(.01, 1.1, 10)
KRR	'alpha': np.logspace(-15, 2, 20),<br>'gamma': np.logspace(-14, -1, 20),<br>'kernel' : ['rbf']
SVR	'C': np.logspace(-1, 4, 20),<br>'epsilon': np.logspace(-2, 2, 20)
RF	'n_estimators': np.linspace(5, 100, 50).astype('int')
Lasso	'alpha': np.logspace(-2, 6, 100)
kNN	'n_neighbors': np.linspace(2,20,18).astype('int')
GBoost	'n_estimators': np.linspace(5, 350, 100).astype('int')
Ridge	'alpha': np.logspace(-6, 6, 150)

Supplementary Table S4. Crystal data and structure refinement for [Hdabco]ClO_4 at 298 K.

Empirical formula	$C_6H_{13}ClN_2O_4$
Temperature /K	298
Crystal system	Orthorhombic
Space group	$Pm2_1n$
Point Group	m2m
General multiplicity	4
a / Å	8.87161
b / Å	9.75012
c / Å	5.35338
α/ º	90
β / º	90
γ / º	90
Volume / $Å^3$	463.0631
Rwp	15.5
Rp	21.9
Chi2:	3.60

Supplementary Table S5. Atom parameters for [Hdabco]ClO_4 at 298 K.

Name	x	y	z	Multiplicity
C1	0.00000	0.45583	0.60183	2
C2	0.00000	0.49907	0.38853	2
C3	0.12749	0.66745	0.65255	4
C4	0.13860	0.68802	0.37158	4
Cl	0.00000	0.04198	-0.01204	2
H1	0.00000	0.52860	0.88788	2
H1A	-0.05763	0.37322	0.77592	4
H2A	0.11694	0.49625	0.37130	4
H3A	0.23031	0.67215	0.78103	4
H3B	0.18569	0.76747	0.73694	4
H4A	0.12118	0.78935	0.28019	4
H4B	0.24530	0.65665	0.30723	4
N1	0.00000	0.62096	0.76603	2
N2	0.00000	0.61193	0.28486	2
O1	0.00000	0.19434	-0.35321	2
O2	0.14225	-0.02118	0.00277	4
O3	0.00000	0.17984	-0.03208	2

Supplementary Table S6. Crystal data and structure refinement for $[Hdabco]ClO_4$ at 360 K.

Empirical formula	$C_6H_{13}ClN_2O_4$
Temperature /K	360
Crystal system	Orthorhombic
Space group	$Pm2_1n$
Point Group	m2m
General multiplicity	4
a / Å	9.0058
b / Å	9.7312
c / Å	5.3669
α/ º	90
β / º	90
γ / º	90
Volume / Å^3	470.3389
Rwp	17.8
Rp	26.3
Chi2:	4.2

Supplementary Table S7. Atom parameters for $[Hdabco]ClO_4$ at 360 K.

Name	x	y	z	Multiplicity
C1	0.00000	0.49629	0.64981	2
C2	0.00000	0.47233	0.39288	2
C3	0.10624	0.66537	0.64488	4
C4	0.13836	0.69143	0.38043	4
Cl	0.00000	0.04767	-0.01015	2
H1	0.00000	0.62084	0.91720	2
H1A	-0.06587	0.35654	0.73494	4
H2A	0.16183	0.46590	0.38049	4
H3A	0.22205	0.61729	0.80440	4
H3B	0.14148	0.81007	0.76892	4
H4A	0.07815	0.78976	0.25354	4
H4B	0.22174	0.69427	0.28280	4
N1	0.00000	0.62364	0.76564	2
N2	0.00000	0.61437	0.28223	2
O1	0.00000	0.06391	-0.21058	2
O2	0.13155	-0.01133	0.04884	4
O3	0.00000	0.19404	0.06423	2

Supplementary Table S8. Crystal data and structure refinement for $[Hdabco]ClO_4$ at 370 K.

Empirical formula	$C_6H_{13}ClN_2O_4$
Temperature /K	370
Crystal system	Orthorhombic
Space group	$Pm2_1n$
Point Group	m2m
General multiplicity	4
a / Å	9.0481
b / Å	9.7302
c / Å	5.3745
α/ º	90
β / º	90
γ / º	90
Volume / $Å^3$	473.1734
Rwp	17.2
Rp	24.6
Chi2:	3.4

Supplementary Table S9. Atom parameters for $[Hdabco]ClO_4$ at 370 K.

Name	x	y	z	Multiplicity
C1	0.00000	0.49189	0.61671	2
C2	0.00000	0.55641	0.33759	2
C3	0.11884	0.70052	0.66375	4
C4	0.14509	0.72714	0.41981	4
Cl	0.00000	0.06989	-0.03115	2
H1	0.00000	0.54784	0.88202	2
H1A	-0.33545	0.44492	0.63927	4
H2A	0.06402	0.35602	0.22016	4
H3A	0.22839	0.62259	0.74544	4
H3B	0.14535	0.80952	0.68735	4
H4A	0.08245	0.81069	0.28134	4
H4B	0.22795	0.70282	0.34678	4
N1	0.00000	0.62105	0.77053	2
N2	0.00000	0.64599	0.27326	2
O1	0.00000	0.16128	-0.16241	2
O2	0.11777	0.02667	0.06240	4
O3	0.00000	0.23559	0.13152	2

Supplementary Table S10. Crystal data and structure refinement for [Hdabco]ClO_4 at 375 K.

Empirical formula	$C_6H_{13}ClN_2O_4$
Temperature /K	375
Crystal system	Orthorhombic
Space group	$Pm2_1n$
Point Group	m2m
General multiplicity	4
a / Å	9.0521
b / Å	9.7053
c / Å	5.3661
α/ º	90
β / º	90
γ / º	90
Volume / $Å^3$	471.4283
Rwp	17.5
Rp	26.3
Chi2:	3.4

Supplementary Table S11. Atom parameters for [Hdabco]ClO_4 at 375 K.

Name	x	y	z	Multiplicity
C1	0.00000	0.51796	0.65491	2
C2	0.00000	0.52860	0.35432	2
C3	0.12744	0.69208	0.62420	4
C4	0.13460	0.71819	0.41524	4
Cl	0.00000	0.07746	-0.01735	2
H1	0.00000	0.80105	0.84223	2
H1A	-0.07472	0.38574	0.68564	4
H2A	0.15136	0.54248	0.08414	4
H3A	0.25838	0.72638	0.75908	4
H3B	0.16203	0.78872	0.77512	4
H4A	0.11968	0.80627	0.26275	4
H4B	0.22738	0.67674	0.31907	4
N1	0.00000	0.65420	0.79329	2
N2	0.00000	0.64637	0.27561	2
O1	0.00000	0.15479	-0.15875	2
O2	0.13228	0.01238	0.03251	4
O3	0.00000	0.20225	0.12040	2

Supplementary Table S12. Crystal data and structure refinement for [Hdabco]ClO_4 at 380 K.

Empirical formula	$C_6H_{13}ClN_2O_4$	$C_6H_{13}ClN_2O_4$
Temperature /K	380	380
Crystal system	Tetragonal	Orthorhombic
Space group	P4/nmm	$Pm2_1n$
Point Group	4/mmm	m2m
General multiplicity	16	4
a / Å	9.44316	9.07880
b / Å	9.44316	9.71960
c / Å	5.36862	5.37953
α/ º	90	90
β / º	90	90
γ / º	90	90
Volume / $Å^3$	478.7377	474.7015
Density/g·cm^{-3}	1.323	1.396
Weight fractions	51.15	48.85
Rwp	16.2	
Rp	16.2	
Chi2:	2.91	

Supplementary Table S13. Atom parameters for paraelectric [Hdabco]ClO_4 at 380 K.

Name	x	y	z	Multiplicity
C1	0.25000	0.25000	1.25010	2
C2	0.14560	0.35420	0.88630	8
C3	0.22520	0.39070	1.14400	16
C4	0.20540	0.38750	0.87620	16
Cl	-0.25000	0.25000	0.54960	4
H1	0.25010	0.25000	1.43800	2
H1A	0.24980	0.24990	0.60920	2
H2A	0.13500	0.45350	1.19140	16
H3A	0.20610	0.47670	1.24550	16
H3B	0.10650	0.39320	0.91130	8
H4A	0.05350	0.32600	0.82550	16
H4B	0.14470	0.35520	1.17020	8
N1	0.25000	0.25000	0.79720	2
N2	-0.37000	0.25000	0.43800	8
O1	-0.14050	0.14050	0.50000	8
O2	-0.25000	0.25000	0.80000	4
O3	-0.14100	0.25000	0.56000	8

Supplementary Table S14. Atom parameters for ferroelectric [Hdabco]ClO_4 at 380K.

Name	x	y	z	Multiplicity
C1	0.00000	0.46197	0.57872	2
C2	0.00000	0.45617	0.43175	2
C3	0.11968	0.67666	0.71469	4
C4	0.12495	0.70615	0.39069	4
Cl	0.00000	0.04164	-0.06484	2
H1	0.00000	0.65835	0.77445	2
H1A	-0.22298	0.31146	0.83717	4
H2A	0.42266	0.56381	0.83440	4
H3A	0.46288	1.21381	1.92036	4
H3B	0.08370	0.70470	0.77380	4
H4A	0.04186	0.76460	0.35549	4
H4B	0.21912	0.66522	0.76690	4
N1	0.00000	0.65575	0.83977	2
N2	0.00000	0.66055	0.34489	2
O1	0.00000	0.71113	0.15479	2
O2	0.22149	-0.02847	0.14153	4
O3	0.00000	0.15286	0.00035	2

Supplementary Table S15. Crystal Data and Structure Refinement for [Hdabco]ClO_4 at 390K

Empirical formula	$C_6H_{13}ClN_2O_4$
Temperature /K	390
Crystal system	Tetragonal
Space group	P4/nmm
Point Group	4/mmm
General multiplicity	16
a / Å	9.4512
b / Å	9.4512
c / Å	5.3710
α/ º	90
β / º	90
γ / º	90
Volume / $Å^3$	479.7701
Rwp	24
Rp	33.9
Chi2:	5.7

Supplementary Table S16. Atom parameters for $[Hdabco]ClO_4$ at 390K.

Name	x	y	z	Multiplicity
C1	0.25000	0.25000	1.25220	2
C2	0.13970	0.36030	0.88210	8
C3	0.22380	0.38720	1.13400	16
C4	0.19950	0.37540	0.86760	16
Cl	-0.25000	0.25000	0.55230	4
H1	0.25000	0.25000	1.44080	2
H1A	0.25000	0.25000	0.60890	2
H2A	0.13480	0.45190	1.17930	16
H3A	0.20670	0.47640	1.22720	16
H3B	0.10120	0.39880	0.90520	8
H4A	0.04660	0.31310	0.83120	16
H4B	0.14370	0.35630	1.16680	8
N1	0.25000	0.25000	0.79760	2
N2	-0.36800	0.25000	0.42200	8
O1	-0.14010	0.14010	0.50000	8
O2	-0.25000	0.25000	0.80200	4
O3	-0.12900	0.25000	0.55200	8

Supplementary Reference

1 Bicerano, J. *Prediction of polymer properties*. (cRc Press, 2002).

2 Wold, S., Sjöström, M. & Eriksson, L. PLS-regression: a basic tool of chemometrics. *Chemometrics Intellig. Lab. Syst.* **58**, 109-130 (2001).

3 Tenenbaum, J. B., Silva, V. d. & Langford, J. C. A global geometric framework for nonlinear dimensionality reduction. *Science* **290**, 2319-2323 (2000).

4 Balasubramanian, M. & Schwartz, E. L. The isomap algorithm and topological stability. *Science* **295**, 7-7 (2002).

5 Srinivasan, S. *et al.* Mapping Chemical Selection Pathways for Designing Multicomponent Alloys: an informatics framework for materials design. *Sci. Rep.* **5**, 1-8 (2015).

6 Eriksson, L., Byrne, T., Johansson, E., Trygg, J. & Vikström, C. *Multi-and megavariate data analysis basic principles and applications*. Vol. 1 (Umetrics Academy, 2013).

7 Broderick, S. & Rajan, K. Informatics derived materials databases for multifunctional properties. *Science and technology of advanced materials* (2015).

8 Tang, Y.-Y. *et al.* Ultrafast polarization switching in a biaxial molecular ferroelectric thin film:[Hdabco] ClO4. *J. Am. Chem. Soc.* **138**, 15784-15789 (2016).

9 Pramchu, S., Jaroenjittichai, A. P. & Laosiritaworn, Y. Phonon and phonon-related properties of MgSiN2 and MgGeN2 ceramics: First principles studies. *Ceram. Int.* **43**, S444-S448 (2017).

10 Peng, J., Najmaei, S., Dubey, M. & Chung, P. W. Dominant ZA phonons and thermal carriers in HfS2. *J. Appl. Phys.* **126**, 164302 (2019).

11 Gonze, X. & Lee, C. Dynamical matrices, Born effective charges, dielectric permittivity tensors, and interatomic force constants from density-functional perturbation theory. *Phys. Rev. B* **55**, 10355 (1997).
