# Supplementary material for: Releasing chemical energy in spatially programmed ferroelectrics
Source: Nat Commun. 2022 Nov 15;13:6959. doi: 10.1038/s41467-022-34819-z (PMC9666659; doi:10.1038/s41467-022-34819-z)
Supplement: Supplementary file 2 — Description of Additional Supplementary Files [file 41467_2022_34819_MOESM2_ESM.pdf]

Description of Additional Supplementary Files

Supplementary Movie 1. The overall process of printing energetic molecular ferroelectric [Hdabco]ClO4 through syringe-based extrusion

Supplementary Movie 2. Decomposition of 3D printed [Hdabco]ClO4 with different weight ratio
