# Supplementary material for: Releasing chemical energy in spatially programmed ferroelectrics
Source: Nat Commun. 2022 Nov 15;13:6959. doi: 10.1038/s41467-022-34819-z (PMC9666659; doi:10.1038/s41467-022-34819-z)
Supplement: Supplementary file 5 — Lasing Reporting Summary [file 41467_2022_34819_MOESM5_ESM.pdf]

nature research

Corresponding author(s): Shenqiang Ren

Lasing Reporting Summary

Nature Research wishes to improve the reproducibility of the work that we publish. This form is intended for publication with all accepted papers reporting claims of lasing and provides structure for consistency and transparency in reporting. Some list items might not apply to an individual manuscript, but all fields must be completed for clarity.

For further information on Nature Research policies, including our data availability policy, see Authors & Referees.

ü Experimental design

Please check: are the following details reported in the manuscript?

1. Threshold

Plots of device output power versus pump power over a wide range of values indicating a clear threshold

- [ ] Yes
- [x] No

The laser energy was fixed for the measurements reported in the manuscript, as described in the cited references on LASEM. (Propellants Explos. Pyrotech. 40, 674-681 (2015).)

2. Linewidth narrowing

Plots of spectral power density for the emission at pump powers below, around, and above the lasing threshold, indicating a clear linewidth narrowing at threshold

- [ ] Yes
- [x] No

In this study, the laser was run at full power only for LASEM (well above the lasing threshold, and not relevant to include the linewidth narrowing).

Resolution of the spectrometer used to make spectral measurements

- [x] Yes
- [ ] No

Included in the experimental description of LASEM measurements in the manuscript.

3. Coherent emission

Measurements of the coherence and/or polarization of the emission

- [ ] Yes
- [x] No

The coherence and polarization does not influence the ablation event. The LASEM measurements in this study are not relevant to the the coherence and polarization.

4. Beam spatial profile

Image and/or measurement of the spatial shape and profile of the emission, showing a well-defined beam above threshold

- [ ] Yes
- [x] No

The LASEM measurements don't depend on the spatial shape and profiles of the emission (the beam shape is roughly Gaussian like most standard Nd:YAG lasers).

5. Operating conditions

Description of the laser and pumping conditions
Continuous-wave, pulsed, temperature of operation

- [x] Yes
- [ ] No

The laser condition is included in experimental description of LASEM measurements.

Threshold values provided as density values (e.g. W cm^{-2} or J cm^{-2}) taking into account the area of the device

- [x] Yes
- [ ] No

The threshold values is provided as density values in the experimental description of LASEM measurements.

6. Alternative explanations

Reasoning as to why alternative explanations have been ruled out as responsible for the emission characteristics
e.g. amplified spontaneous, directional scattering; modification of fluorescence spectrum by the cavity

- [ ] Yes
- [x] No

Laser ablation and laser-induced plasma formation is well-documented in the literature; the cited reference (Propellants Explos. Pyrotech. 40, 674-681 (2015).) on LASEM explains the influences on the laser-induced shock velocities.

7. Theoretical analysis

Theoretical analysis that ensures that the experimental values measured are realistic and reasonable
e.g. laser threshold, linewidth, cavity gain-loss, efficiency

- [x] Yes
- [ ] No

Laser-induced shock velocities for military explosives with known properties were used develop the correlation to explosive performance (Fig 5b); the results agree well with theoretically predicted detonation velocities. The exact calibration is system-dependent.

8. Statistics

Number of devices fabricated and tested

- [ ] Yes
- [x] No

A standard commercial off the shelf industrial-grade laser was used for the LASEM study. Not relevant to include the number of devices fabricated and tested.

Statistical analysis of the device performance and lifetime (time to failure)

- [ ] Yes
- [x] No

A standard commercial off the shelf industrial-grade laser was used for the LASEM study. Not relevant to include the statistical analysis of the lifetime.
